# Human talar ontogeny: Insights from morphological and trabecular changes during postnatal growth

**DOI:** 10.1002/ajpa.24596

**Published:** 2022-08-06

**Authors:** Carla Figus, Nicholas B. Stephens, Rita Sorrentino, Eugenio Bortolini, Simona Arrighi, Federico Lugli, Giulia Marciani, Gregorio Oxilia, Matteo Romandini, Sara Silvestrini, Fabio Baruffaldi, Maria Giovanna Belcastro, Federico Bernardini, Igor Erjavec, Anna Festa, Tamás Hajdu, Orsolya Mateovics‐László, Mario Novak, Ildikó Pap, Tamás Szeniczey, Claudio Tuniz, Timothy M. Ryan, Stefano Benazzi

**Affiliations:** ^1^ Department of Cultural Heritage University of Bologna Ravenna Italy; ^2^ Department of Anthropology Pennsylvania State University University Park Pennsylvania USA; ^3^ Department of Biological, Geological and Environmental Sciences – Bigea University of Bologna Bologna Italy; ^4^ Human Ecology and Archaeology (HUMANE) Barcelona Spain; ^5^ Laboratory of Medical Technology IRCCS Istituto Ortopedico Rizzoli Bologna Italy; ^6^ Department of Humanistic Studies Università Ca'Foscari Venezia Italy; ^7^ Multidisciplinary Laboratory Abdus Salam International Centre for Theoretical Physics Trieste Italy; ^8^ Laboratory for Mineralized Tissue Centre for Translational and Clinical Research Zagreb Croatia; ^9^ Department of Biological Anthropology, Institute of Biology, Faculty of Science Eötvös Loránd University Budapest Hungary; ^10^ Archaeological Heritage Directorate Hungarian National Museum Budapest Hungary; ^11^ Centre for Applied Bioanthropology Institute for Anthropological Research Zagreb Croatia; ^12^ Department of Anthropology Hungarian Natural History Museum Budapest Hungary; ^13^ Department of Biological Anthropology, Institute of Biology, Faculty of Science and Informatics Szeged University Szeged Hungary; ^14^ Centre for Archaeological Science University of Wollongong Wollongong Australia

**Keywords:** bipedal locomotion, geometric morphometrics, human growth, ontogeny, trabecular morphology

## Abstract

**Objectives:**

The study of the development of human bipedalism can provide a unique perspective on the evolution of morphology and behavior across species. To generate new knowledge of these mechanisms, we analyze changes in both internal and external morphology of the growing human talus in a sample of modern human juveniles using an innovative approach.

**Materials and Methods:**

The sample consists of high‐resolution microCT scans of 70 modern juvenile tali, aged between 8 postnatal weeks and 10 years old, from a broad chronological range from Middle/Late Neolithic, that is, between 4800 and 4500 BCE, to the 20th century. We applied geometric morphometric and whole‐bone trabecular analysis (bone volume fraction, degree of anisotropy, trabecular number, thickness, and spacing) to all specimens to identify changes in the external and internal morphology during growth. Morphometric maps were also generated.

**Results:**

During the first year of life, the talus has an immature and globular shape, with a dense, compact, and rather isotropic trabecular architecture, with numerous trabeculae packed closely together. This pattern changes while children acquire a more mature gait, and the talus tends to have a lower bone volume fraction, a higher anisotropy, and a more mature shape.

**Discussion:**

The changes in talar internal and external morphologies reflect the different loading patterns experienced during growth, gradually shifting from an “unspecialized” morphology to a more complex one, following the development of bipedal gait. Our research shows that talar plasticity, even though genetically driven, may show mechanical influences and contribute to tracking the main locomotor milestones.

## 1 INTRODUCTION

Among extant primates, committed bipedalism is unique to our species (*Homo sapiens*), and debates about its evolutionary history are a central topic in the field of paleoanthropology. Even if many aspects of our anatomy contribute to this form of locomotion, researchers often focus on the skeletal morphology of the lower limb in adult bone (DeSilva, 2009; DeSilva et al., 2013; Frelat et al., 2012, 2017; Lewton & Scott, 2017; Sorrentino et al., 2021; Sorrentino, Stephens, et al., 2020; Tsegai et al., 2017; Turley et al., 2011, 2015; Turley & Frost, 2013, 2014a; Zeininger et al., 2016), with particular emphasis on the functional morphology of the talus (DeSilva et al., 2019; Harcourt‐Smith & Aiello, 2004; McNutt et al., 2018; Sorrentino, Carlson, et al., 2020). Fewer studies, though, investigate how bone adapts to bipedal locomotion during human development (Colombo et al., 2019; Figus et al., 2022; Gosman & Ketcham, 2009; Raichlen, 2005; Raichlen et al., 2015; Ryan & Krovitz, 2006; Saers et al., 2020; Shapiro & Raichlen, 2006). The talus is of paramount importance as it unites the foot and the leg, and receives the weight of the body from the tibia. It plays an essential role in distributing the weight of the body during walking and standing, and its center has been recognized as a fundamental region for force distribution (Cunningham et al., 2016). Consequently, the talus plays a key role in the different stages of human locomotion, from crawling, to initial bipedal acquisition, to full striding bipedalism (Hellier & Jeffery, 2006). The loading patterns of the talus during early childhood are irregular and become more predictable as children grow, to the extent that they potentially capture the shift from unstable to stable locomotion (Raichlen et al., 2015; Saers et al., 2020). Unfortunately, however, there is limited research on talus growth (Cunningham et al., 2016; Figus et al., 2022; Hellier & Jeffery, 2006; Scheuer & Black, 2004; Turley et al., 2018).

Given the complexity of the ontogenetic development, and the paucity of studies about the human talus, this work aims to: (1) highlight the ontogenetic morphological changes from birth to about 10 years of age in the human talus; (2) quantify the trabecular bone properties from early infancy to pre‐pubertal age, to better understand how the different factors (e.g., mechanical forces, activity level, genetics, and physiological changes) act on the human talus. This work will ultimately shed light on adult bone morphology and the relationship between form and function.

### 1.1 The role of the talus in the human ankle

Due to the derived anatomy of the human foot, for example, a stiff longitudinal arch, adducted hallux, short toes, and complex talar structure which, in synergy with muscles and ligaments, significantly participate in forming a stiff lever (Farris et al., 2019), humans perform bipedal locomotion with relative low energetic efforts. The skeletal morphology of the foot is central to reconstructing the evolutionary history of bipedalism (Prang, 2016; Sorrentino, Carlson, et al., 2020; Sorrentino et al., 2021; Su & Carlson, 2017; Turley et al., 2015). The human talus occupies a pivotal role in forming the ankle (or talocrural joint). It is often described as a “carpenter's mortise and tenon” joint (Aiello & Dean, 1992), where the lateral malleolus of the fibula and medial malleolus of the tibia, together with the lower articular tibial surface, are the mortise, and the talus is the tenon. The ankle joint participates in plantar‐ and dorsal‐flexion and contributes to other foot movements such as abduction‐adduction and inversion‐eversion (Aiello & Dean, 1992; Griffin et al., 2015). The ankle is held together by a set of small ligaments on the lateral side and a strong deltoid ligament on the medial side, which help to prevent the displacement of the talus. The talus is a very robust bone, and it is the only talar bone without any muscular or tendinous attachments, though it has extensive ligamentous attachments. This means that talar movements are passive, resulting from forces acting on the talus after being transmitted from other bones (Aiello & Dean, 1992; Parr et al., 2014). Its main role is to receive the load from the leg, then transmits the weight posteriorly to the calcaneus, on which the body of the talus rests, and anteriorly to the navicular through the spring ligament, which is an osseo‐ligament suspended across a gap between the sustentaculum tali and the navicular bone (Moore & Dalley, 2018). Most of its surface is covered with hyaline cartilage (Athanasiou et al., 1995). The talar body bears the trochlea superiorly and narrows into a posterior process that features a groove for the tendon of the *flexor hallucis longus*, flanked by a prominent lateral tubercle and a less prominent medial tubercle. During walking, due to both the rolling of the tibia on the trochlea and the changes in talus position, it is exposed to varying loads (Pal & Routal, 1998).

### 1.2 Human gait cycle and its development

The modern human gait cycle is a complex set of motions that broadly alternate between the stance phase and swing phase, during which the human foot acts as a shock absorber at heel strike and as a rigid lever at toe‐off, thanks to the windlass mechanism (Griffin et al., 2015). Bodyweight is transferred from 1 foot to the other in a sideways manner. The stance phase begins at the initial contact of the heel with the ground (i.e., “heel strike”), with the knee fully extended and the foot dorsiflexed, allowing the calcaneus to touch the ground and absorb the shock first as the foot begins to assume the body's full weight (Moore & Dalley, 2018). Forces are transmitted from the ground through the lateral side of the foot as it rolls to a flat position, and enters the midstance phase, where the total body weight is borne by the planted foot. From here the foot, acting as a lever, passes the forces medially and over to the ball of the foot, while the strong plantar flexor muscles contract, pushing the forefoot down to generate a propulsive force that results in a push‐off stage (Hennig & Rosenbaum, 1991). During this phase of the gait cycle, the toes flex to grip the ground, augmenting the earlier push‐off with a “toe‐off” that ends when the hallux leaves the ground. Swing phase begins with the knee and hip bent to permit the leg to move forward as the other foot touches down.

The acquisition of bipedalism is a complex process that involves both the musculoskeletal and neuromuscular systems (Chagas et al., 2006). Some important morphological differences between juveniles and adults may explain some of the differences between immature and mature locomotion. At birth, the skeletal structure is mainly cartilaginous, and the ossification and developmental processes in the foot occur mostly during the first 6 years of life (Hallemans et al., 2003). Talar ossification starts around 6–7 months of age typically from one center (Gray et al., 1957). Even considering that individual variability and cultural influences may affect the timing of locomotor development, which is not linear and is also affected by different childrearing practices (Adolph & Franchak, 2017; Cowgill & Johnston, 2018), it is possible to recognize some developmental milestones. Children start trying to sit upright, with the head well balanced over the neck and shoulder at about 6 months. They may start crawling on all four limbs at about 9 months and gradually stand in an upright position and start walking unaided at about 12 months, acquiring the basic skills to perform stable locomotion during the first months of practice (Chagas et al., 2006). Toddlers often practice an “on all‐four” quadrupedal behavior (e.g., using hands and knees, rarely on hands and feet) (Abitbol, 1993). The use of these two locomotor behaviors varies between individuals and are often intermingled for a time (Abitbol, 1993). Children may also skip or return to some phases (Adolph & Franchak, 2017).

During the first 12–18 months, children grow at a fast pace, with the legs growing faster than the trunk (Adolph & Avolio, 2000). At ~1 year of age, at the time of the onset of bipedal locomotion, the primary ossification centers of the talus, calcaneus, and some of the phalanges are still surrounded by cartilaginous tissue (Hallemans et al., 2003). Children usually have a wider walking base and produce short steps at a slow pace (Levine, 2002). During the earliest locomotor phase, steps are highly irregular with high step‐to‐step variability, which is helpful to maintain balance while the muscular control system is still immature (Adolph et al., 2003; Clark et al., 1988; Hallemans et al., 2003; Sutherland, 1997; Thelen, 2015). In this phase, the vastus medialis and gluteus maximus play a pivotal role in balance control (Okamoto et al., 2003). Mediolateral trunk oscillations are significantly higher in new walkers as well reflecting variation in joint angles from the ankle through the pelvis (Breniere & Bril, 1988; Raichlen et al., 2015). Infants have a dominance of hip and knee extending moments during stance, with no active push‐off forces at the ankle joint (Hallemans et al., 2005).

Due to the weakness of the tibialis anterior which does not permit the dorsiflexion of the foot during the early phase of locomotor maturation, the “heel‐to‐toe” roll‐over pattern is absent (Hallemans et al., 2006) and the entire foot still contacts the ground to increase the base of support (Zeininger et al., 2018). The immature longitudinal arch, which is covered by a protective fat pad (Cunningham et al., 2016), contributes to the infant's physiological flat foot. This has been thought to be a contributing factor in the difference between mature and immature gait patterns (Hallemans et al., 2003). After three to 6 months of unassisted bipedal walking, steps become longer, narrower, and straighter, with a more consistent pattern, and less step‐to‐step variability (Adolph et al., 2003; Forssberg, 1985; Sutherland et al., 1980), and improved balance. The transfer of the center of pressure (COP) from the lateral to medial side develops around 18 months (Bertsch et al., 2004), while the heel strike pattern does not develop until around 18–24 months on average (Zeininger et al., 2018).

Between 2 and 3 years of age changes in development occur more rapidly (Preis et al., 1997). The valgus inclination of the ankle, which is present at birth, changes into a neutral position by age 3 (Hallemans et al., 2003). When the longitudinal arch is developing, peak plantar pressures on the fore‐ and hind‐foot increase while pressure is reduced in the midfoot reaching adult levels between 5 and 6 years (Bertsch et al., 2004; Zeininger, 2013). Initially, when the roll‐off is absent or still immature, the medial areas of the plantar foot surface show a great contribution to load‐bearing, and during the contact time, the entire foot is touching the ground (Hallemans et al., 2003). Contact areas under the immature foot are small since the load is distributed evenly over the entire plantar surface, contributing to the generation of propulsive motion (Hallemans et al., 2003). Gradually, the pattern changes, and the load shifts toward the lateral side of the foot. A reduction in peak pressures under the hallux is followed by an increase in pressure under the lateral side of the foot (Hallemans et al., 2003). With the maturation of the longitudinal arch, which avoids the over‐loading of the midfoot, the foot structure becomes more similar to the adult one. A mature form of bipedalism fully develops around 7 or 8 years of age (Bernstein et al., 2000; Breniere & Bril, 1988; Bril & Ledebt, 1998; Sutherland et al., 1980).

### 1.3 Bone (re)modeling during growth and predictions

Both cortical and trabecular bone are highly responsive to the loading environments (Barak, 2019; Barak et al., 2011; Carlson & Judex, 2007; Pontzer et al., 2006; Ruff & Hayes, 1982; Ruff et al., 2006; Wolff, 1892), with the rate of bone (re)modeling during the first 2 years of life being higher than in adults, as also suggested by numerous cross‐sectional studies (Chevalier et al., 2021; Gosman & Ketcham, 2009; Milovanovic et al., 2017; Raichlen et al., 2015; Ryan & Krovitz, 2006; Ryan et al., 2017; Saers et al., 2020). In particular, studies on the tibia (Gosman & Ketcham, 2009; Raichlen et al., 2015), femur (Milovanovic et al., 2017; Ryan & Krovitz, 2006), vertebrae (Acquaah et al., 2015), talus (Figus et al., 2022), and calcaneus (Saers et al., 2020) describe a general typical pattern in which, around birth, high bone volume fraction (BV/TV) corresponds to high trabecular number (Tb.N) and low trabecular thickness (Tb.Th) and spacing (Tb.Sp). This contributes to the “dense” aspect of the early postnatal trabecular bone. During the first year of life, a drastic reduction in BV/TV is accompanied by a decrease in Tb.N. After 1 year of age, BV/TV gradually increases again, along with an increase in Tb.Th (Ryan et al., 2017). Nevertheless, the degree of anisotropy (DA) shows a different and region‐dependent pattern. Around birth, the architecture is rather isotropic in vertebrae, calcaneus, and talus (Acquaah et al., 2015; Figus et al., 2022; Saers et al., 2020), followed by a gradual increase in DA during the first year of life. A different pattern has been noted in the femur, tibia, and humerus (Gosman & Ketcham, 2009; Milovanovic et al., 2017; Ryan & Krovitz, 2006), where an anisotropic structure in the youngest individuals is followed by a decrease in fabric structure during the first year of age. At that stage, DA started increasing, together with the development of a more consistent locomotor pattern. More recent studies (Chevalier et al., 2021; Ryan et al., 2017), however, suggest that early bone development may be linked to a physiological phase rather than to functional loading: for example, for Acquaah et al. (2015) a large amount of bone deposited during the fetal period in the vertebral column may also serve as a calcium reservoir after birth, which is dramatically reabsorbed during the first postnatal months.

Based on previous ontogenetic studies (Acquaah et al., 2015; Chevalier et al., 2021; Colombo et al., 2019; Figus et al., 2022; Hellier & Jeffery, 2006; Raichlen et al., 2015; Ryan et al., 2017; Saers et al., 2020), we predict that talar internal and external morphology will reflect the change in loading associated with the acquisition of bipedal gait. The orientation and shape of the articular facets are expected to change with increasing body mass to efficiently distribute the forces in a medial direction as gait matures. This will be reflected in a more medially oriented head and neck, and an increase in the size and curvature of the trochlear surface (Hellier & Jeffery, 2006). Based on previous studies on the trabecular bone during growth (Chevalier et al., 2021; Colombo et al., 2019; Figus et al., 2022; Raichlen et al., 2015; Ryan & Krovitz, 2006; Ryan et al., 2017; Saers et al., 2020), we predict that a relatively isotropic architecture will characterize the talar internal structure, as seen in the calcaneus (Saers et al., 2020) and talus (Figus et al., 2022), in the youngest individuals, with densely packed struts, consisting of a large number of thin trabeculae. BV/TV is expected to decrease during the first year and then increase again after 1 year of age. Following the physiological resorption and increase in mechanical strain, struts are expected to become more anisotropically organized, with thicker and more widely spaced trabeculae, as redundant trabeculae are expected to be reabsorbed. Moreover, we predict that anisotropy will increase from birth, faster after the onset of locomotion, and then the values will rise more slowly in the oldest age groups, as a mature gait has been reached. We also expect to see a higher level of BV/TV and DA in the posterior part of the talar body, in particular in the area of the posterior calcaneal facet and the head, as forces received from the leg are transmitted to the rest of the foot, for example, calcaneus and navicular.

## 2 MATERIALS AND METHODS

The sample consists of 70 juvenile tali aged between 8 postnatal weeks and 10 years old, divided into a control sample of 14 individuals for which information about age, sex, and causes of death was available (F = 8, M = 6; 11 months–11 years) and an archeological sample of 56 individuals (Tables 1 and S1). All tali were selected based on preservation, with a preference for left‐sided elements. In cases where the left was missing/incomplete, right tali were chosen, and digitally mirrored (Islam et al., 2014; Tümer et al., 2019). The control sample refers to the modern (19th–20th) documented skeletal collection, part of the anthropological museum collections of the University of Bologna (Belcastro et al., 2017). The causes of death show that, except for one individual (BO‐14‐M), all the children suffered from acute illnesses, and probably died suddenly, that is, with no influence on the acquisition of the locomotor abilities, for example, weakness due to chronic illnesses.

**TABLE 1:** Study sample

Site	Period	Age‐at‐death range	Number of specimens
Bologna (Italy)	20th Century	11 months–9 years	14
Velia (Italy)	~100–200 CE	0–3 months–9 to 10 years	19
Norris Farms (Illinois, United States)	1300 CE	8 weeks–5 years	13
Beli Manastir (Croatia)	~4800–4500 BCE	2–3.5 years–7 to 9 years	4
Perkáta‐Nyúli dűlő (Hungary)	10th–16th Century	1.5–3 years–7 to 8 years	9
Ilok (Croatia)			
Kraljevića Street	1526–1688 CE	1.5–2 years	1
Krstbajer	1200–1500 CE	2–3 years–5 to 6 years	5
Paks TO‐18 (Hungary)	14th–16th Century	3–5 years–7 to 8 years	5

*Note*: The specimens are ordered by age, from the youngest to the oldest.

The archeological sample consists of individuals from the middle/late Neolithic layers of a site near Beli Manastir, Croatia (*n* = 4, ~4800–4500 BCE) (Los, 2020); the Roman site of Velia, Italy (*n* = 19, ~100–200 CE) (Fiammenghi, 2003; Morel, 2006); the Norris Farms#36 in Illinois, United States (*n* = 13, ~1300 CE) site ascribed to the Oneota culture (Milner et al., 1991; Milner & Smith, 1990); the site of Ilok, Croatia (Krstbajer, *n* = 5, ca 1200–1500 CE; Vlatka Kraljevića Street, *n* = 1, 1526–1688 CE) (Krznar & Rimpf, 2018; Rimpf & Novak, 2020); the sites of Paks‐Cseresznyés (*n* = 5, 14th–16th Centuries) (Hatházi, 2004; László, 2008,  2018; Mesterházy‐Ács, 2015; Szeniczey et al., 2019) and the Cuman settlement from Perkáta‐Nyúli dűlő (*n* = 9, 10th–16th Centuries), in Hungary. In all cases, age at death was estimated based on the assessment of skeletal and dental maturation (Ferembach, 1980; Milner & Smith, 1990; Moorrees et al., 1963a, 1963b; Smith, 1991; Stloukal & Hanáková, 1978).

The sample was subset into four age classes (Table 2) based on previous literature (adapted from Swan et al., 2020) as follows:

- Neonates and infants (0–1 year), which includes infants that were unable to walk independently and those that engaged in a mix of (in)dependent locomotor behaviors (e.g., cruising, crawling, assisted walking).
- Toddlers (1.1–3 years), which includes infants able to walk independently with an immature toddling gait.
- Early childhood (3.1–6 years), which includes infants in an intermediate phase between immature and mature gait.
- Late childhood (6.1–10 years), which includes children who have achieved mature locomotion.

**TABLE 2:** Age classes

Age classes	Group name	Individuals per classes
0–1	Neonates and infants	12
1.1–3	Toddlers	25
3.1–6	Early childhood	21
6.1–10	Late childhood	12
Total		70

### 2.1 Computed tomography

Digital volumes of tali were computed using computed tomographic scans at a voxel size between 0.012 and 0.038 mm. Due to the multinational housing of specimens, tali were scanned at multiple facilities (Tuniz et al., 2013) (see Table 3). Following image acquisition, tomographic scans were reconstructed as 16‐bit TIFF stacks. The resulting volumes were visually inspected with Image J (Schneider et al., 2012), and specimens were excluded from analyses if extensive internal damage or pathology were present. Following this, Avizo 9.3 (Visualization Sciences Group, SAS) was used to crop, resample, and—in cases where heavy sediment or mummified tissues were present—the label field editor was used in conjunction with a Wacom board and paint‐brush to manually remove extraneous material.

**TABLE 3:** MicroCT information

Sample	Facility	Type of scanner	Voxel size (μ)
Bologna	Center for Quantitative Imaging (CQI), Pennsylvania State University, PA (United States)	General Electric v|tome|x L300 nano/microCT	20–38
Norris Farms	Center for Quantitative Imaging (CQI), Pennsylvania State University, PA (United States)	General Electric v|tome|x L300 nano/microCT	12–26
Velia	“Abdus Salam” International Centre of Theoretical Physics (ICTP) in Trieste (Italy)	microfocus X‐ray computed tomography^a^	18–30
	Rizzoli Institute, Bologna, Italy	Skyscan 1072 system (Bruker Corp., Kontich, Belgium).	18
Beli Manastir and Ilok	University of Zagreb, School of Medicine, Zagreb, Croatia	Skyscan 1076 system (Bruker Corp., Kontich, Belgium).	18–29
Paks and Perkata	The Abdus Salam International Centre for Theoretical Physics, Trieste, Italy	microfocus X‐ray computed tomography	18–30

^a^ System specifically designed in collaboration with Elettra Sincrotrone (Trieste) for the study of paleontological and archeological materials (Tuniz et al., 2013).

Segmentation of image volumes was performed using the MIA clustering method (Dunmore et al., 2018), which subdivides volumes into overlapping volumes and uses a K‐means algorithm followed by a fuzzy C‐means algorithm to cluster gray values into user‐determined classes (Bezdek et al., 1987; Dunmore et al., 2018; Dunn, 1973). In cases where the contrast between sediment and bone was poor, a White Top‐hat filter was applied to image volumes to enhance the contrast (Soille, 2000).

### 2.2 Geometric morphometrics analysis

**FIGURE 1:**
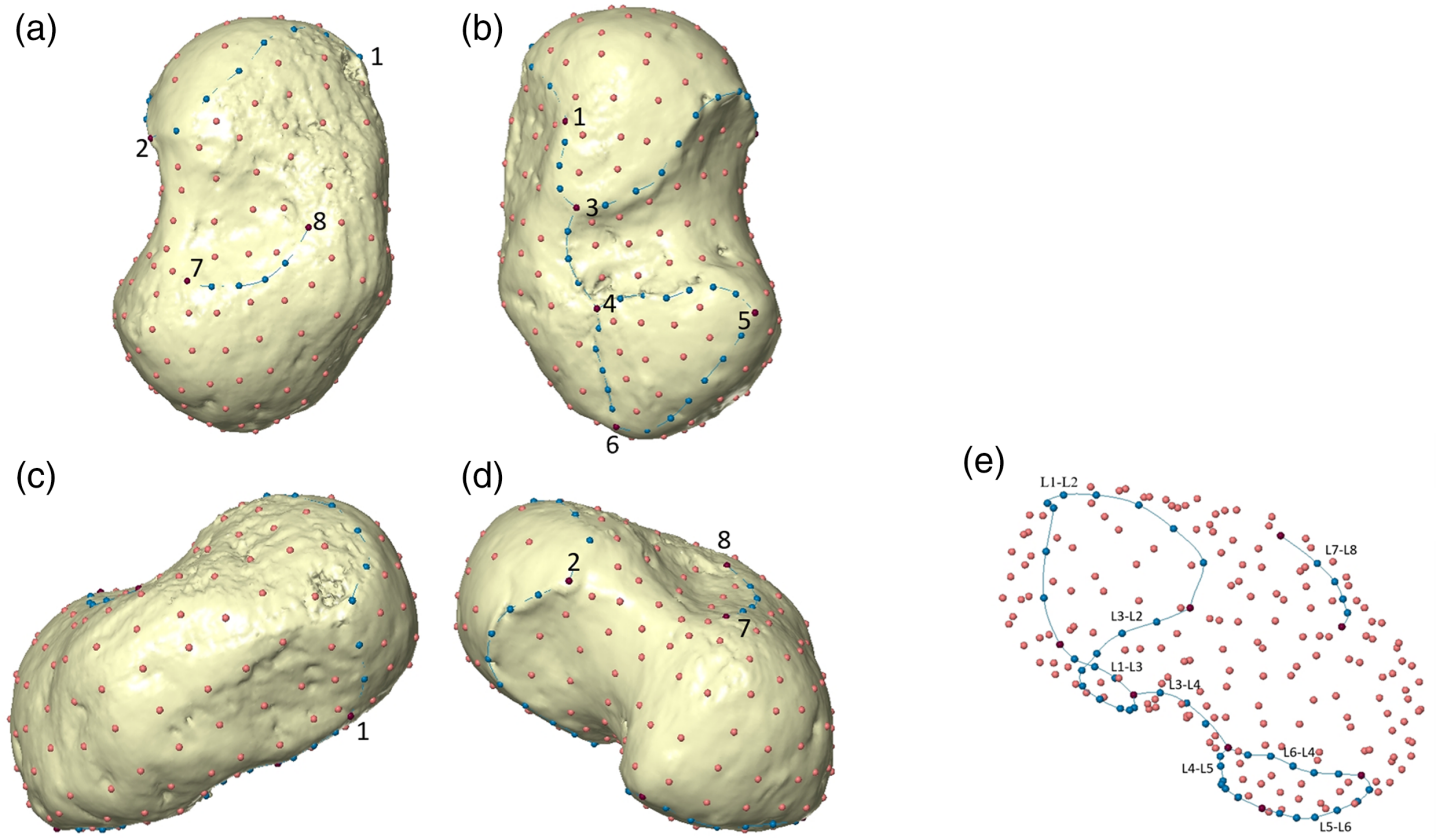
Configuration of (semi)landmarks. (a) Dorsal view; (b) palmar view; (c) medial view; (d) lateral view. Landmarks are represented in purple, curve semilandmarks are in turquoise, and surface semilandmarks are in salmon. (e) Configuration of (semi)landmarks. Curves (turquoise points) are highlighted

A template of 228 (semi)landmarks (8 anatomical landmarks, 45 curve semilandmarks, and 175 surface semilandmarks) was created in Viewbox 4 (dHAL Software), from a 1.9‐year‐old female specimen (BO‐14‐F) (Figure 1, Tables 4 and 5). The (semi)landmark configurations were applied to all of the targets; semilandmarks were then allowed to slide on curves and surfaces to minimize thin‐plate‐spline bending energy (Slice, 2006) between template and targets and to make them geometrically homologous among individuals (Gunz & Mitteroecker, 2013; Mitteroecker et al., 2013). Coordinates were registered with a generalized procrustes analysis (GPA) using the R (R Core Team 2020) package geomorph 3.3.1 (Adams & Otárola‐Castillo, 2013). Size was removed (centroid size, CS = 1) and the targets were translated and rotated to minimize the Procrustes distance between homologous (semi)landmarks. Semilandmarks were then allowed to slide against recursive updates of the Procrustes consensus (Rohlf & Slice, 1990; Slice, 2006). A shape space principal component analysis (PCA) was carried out on the Procrustes coordinates to explore shape variation using the R package Morpho 2.8 (Schlager, 2017). Shapiro Normality Test and Levene test were performed on the first three principal components (PCs), to assess the distribution of the data and its homoscedasticity. Then, based on the fulfillment of the assumptions, the respective parametric (analysis of variance—ANOVA, Tukey's post‐hoc test) or non‐parametric tests (Kruskal–Wallis rank‐sum test, Dunn's test) were performed to find any significant variance between age group means along the first three PCs. Pearson's product–moment correlation (*r*) was performed to assess any size‐related shape variations (i.e., the natural logarithm of the CS). The variations of size and shape were analyzed through PCA in Procrustes form space by adding the natural logarithm of CS as an additional variable to Procrustes shape coordinates (Gunz & Mitteroecker, 2013; Klingenberg, 2016; Mitteroecker et al., 2004). The form space PCA reduces shape variation in a few dimensions while retaining size information (Mitteroecker et al., 2004).

**TABLE 4:** List of anatomical landmarks

Label	Description	Type of landmark
1	Medial point of contact between the navicular and anterior calcaneal facets	II
2	Point of contact between the navicular facet and lateral ridge	II
3	Most posterior point of anterior calcaneal facet	III
4	Most anterior point of posterior calcaneal facet	III
5	Most lateral point of posterior calcaneal facet	III
6	Most posterior point of posterior calcaneal facet	III
7	Most posterior lateral point of the proximal neck (i.e., lateral point of the anterior margin of the trochlea)	III
8	Most posterior medial point of the proximal neck (i.e., medial point of the anterior margin of the trochlea)	III

*Note*: Type of landmarks according to Bookstein (1997).

**TABLE 5:** Semilandmarks

Semilandmarks on curves	*N*
Curve L1–L2: Dorsal border of the head/navicular facet	9
Curve L1–L3: Medial border of anterior calcaneal facet	3
Curve L3–L2: Posterior margin of the anterior‐medial calcaneal facet/head	9
Curve L3–L4: Sulcus tali	3
Curve L4–L5: Anterior border of the posterior calcaneal facet	6
Curve L5–L6: Posterior border of the posterior calcaneal facet	6
Curve L6–L4: Medial border of the posterior calcaneal facet	5
Curve L7–L8: Posterior margin of neck/ anterior margin of the trochlea	4

Semilandmarks on surface	*N*
Neck	10
Head	30
Posterior calcaneal facets	11
Sulcus tali	23
Lateral ridge	10
Lateral side	23
Posterior side and trochlea	33
Medial side	35

### 2.3 Trabecular analyses

**FIGURE 2:**
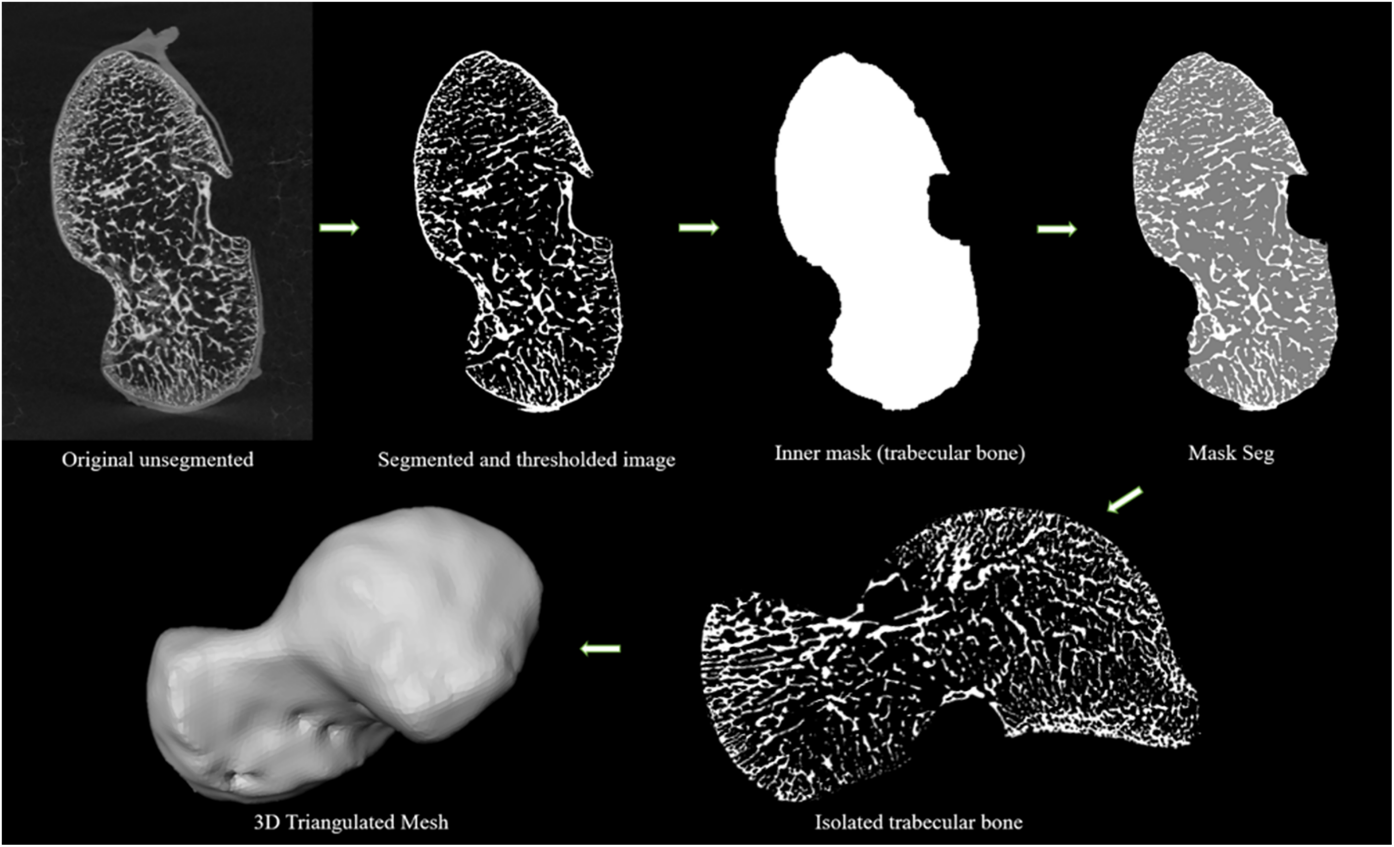
Trabecular quantification workflow. First, the gray value volume is segmented and thresholded. Masks are then generated to separate the cortical and trabecular bone. Finally, a tetrahedral mesh is generated to map the trabecular results onto

Following segmentation, the trabecular and cortical bone were separated following Gross et al. (2014) using Medtool 4.3 (Dr. Pahr Ingenieurs e.U). This is achieved by applying opening and closing filters of varying kernel sizes (3–5 voxels). A filling procedure designed to algorithmically delineate the internal border of the cortical shell was then performed (Pahr & Zysset, 2009). In cases where the cortical bone was too porous, an iterative dilation and erosion cycle was applied to obtain a closed cortical shell. Following this, an outer mask (i.e., external surface) and inner mask (i.e., inner surface) are detracted from the original segmentated image to separate the cortical and trabecular bone (Gross et al., 2014) (see Figure 2). Finally, a tetrahedral mesh of trabecular bone was generated using the computational geometry algorithms library CGAL (www.cgal.org), a mesher that creates a 3D element model using Delaunay triangulation (Delaunay, 1934; Gross et al., 2014; Komza & Skinner, 2019).

BV/TV and DA were quantified on the segmented volume using a 5 mm spherical volume moving along a background grid of 2.5 mm spaced nodes (Gross et al., 2014; Pahr & Zysset, 2009). DA was calculated using the mean intercept length (MIL) approach (Odgaard, 1997), which gave results for first, second, and third eigenvectors and eigenvalues. Then, the fabric DA was calculated as (1‐[eigenvalue3/eigenvalue1]) and scaled between 1 and 0, where 1 indicates a highly anisotropic pattern and 0 a completely isotropic one. The results were then interpolated to the centroid of the elements within the tetrahedral meshes. Colormaps were visualized in Paraview 3.14.1 (Sandia Corporation, Kitware Inc). Mean trabecular thickness (Tb.Th, mm), mean trabecular number (Tb.N), and mean trabecular spacing (Tb.Sp, mm) were calculated (Hildebrand & Rüegsegger, 1997).

To better visualize the differences between groups, 3D statistical comparisons between the two oldest age groups were performed following the Phenotypic PointCloud Analysis protocol (DeMars et al., 2021) for DA and BV/TV. Briefly, trabecular meshes were aligned using a modified version of the MATLAB auto3dgm package (Tingran, Winchester, & Stephens, 2020). The auto3dgm package presents an algorithm that allows the fully automatic placement of correspondence points on digital models. Then, these pseudolandmarks (i.e., landmark‐like points) can be input into standard geometric morphometrics software (Boyer et al., 2015). Here, we used a set of 1200 automatically placed pseudolandmarks. Subsequently, a GPA was carried out using the geomorph 3.3.1 R package (Adams et al., 2018), and we proceeded to find the closest‐to‐mean specimen. This step is necessary to generate a mean mesh by finding the closest‐to‐the‐mean specimens, on which we warped the mean GPA coordinates of all our samples (Stephens et al., 2018). The average trabecular mesh generated was then tetrahedralized with evenly spaced (1.75 mm) points using TetWild (Hu et al., 2020) and vertices were converted to a point cloud. Individual point clouds were then obtained by interpolating BV/TV and DA scalar values to the vertices of the tetrahedral mesh, which were then aligned by applying the auto3dgm transformation matrices followed by a rigid, affine, and deformable alignment using a python implementation of the Coherent Point Drift algorithm (Myronenko & Song, 2010). BV/TV and DA scalar values were linearly interpolated from each individual's pointclouds to the corresponding points in the canonical pointcloud using SciPy (Virtanen et al., 2020a, 2020b) and the mean, standard deviation, and coefficient of variation for each group were mapped onto the average pointcloud, and statistically compared across the sample. The homologous points were compared using a two‐tailed *t* test, with *p* values corrected for multiple comparisons using random field theory, to control for the chance of false positives (Friston, 1995; Worsley et al., 1996, 2009). Results were interactively visualized in Paraview with figures being automatically generated using PyVista (Sullivan & Kaszynski, 2019).

## 3 RESULTS

### 3.1 External morphology

**FIGURE 3:**
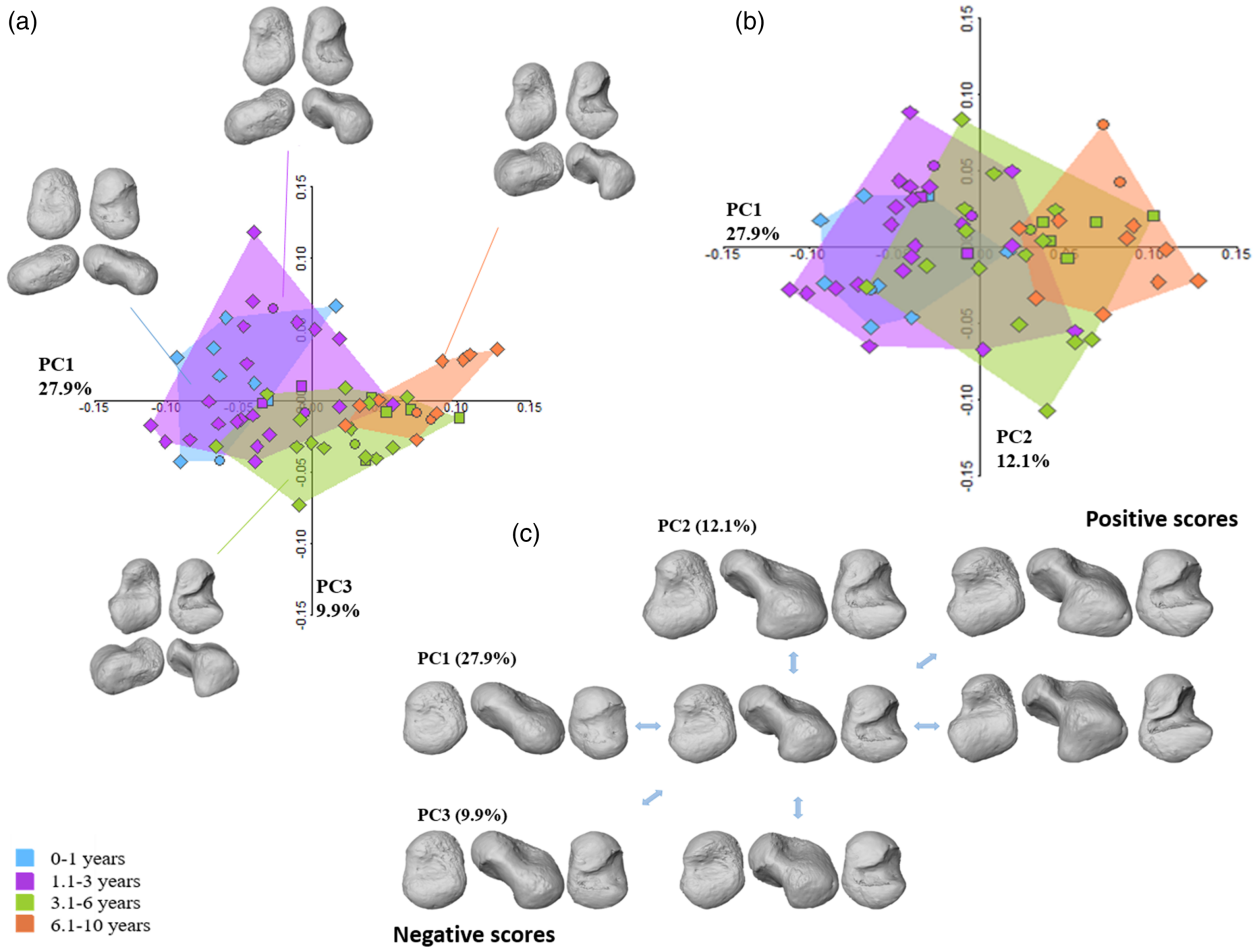
Results from the ontogenetic shape variation of the talus. Principal component analysis (PCA) plot showing PC1 versus PC3 (a) and the mean shape for each age group. Variation among age groups is also represented in a PCA plot showing PC1 versus PC2 (b) whereas extreme shapes along the axis are depicted in (c). Individuals from Bologna, for which sex was known, are represented by circles (males) and squared symbols (female). Triangles represent the archeological samples

Shapiro–Wilk normality test shows that the first two PCs are normally distributed (PC1: *W* = 0.97; *p* value = 0.2; PC2: *W* = 0.98, *p* value = 0.7), while PC3 is not normally distributed (*W* = 0.94, *p* value = 0.004). A Levene test supports the homogeneity of variance of the first three PCs, with a *p* value >0.1. The first three PCs account for 50.1% of the total variance (Figure 3a,b), with PC1 explaining 27.9%. The two youngest groups (i.e., 0–1 and 1.1–3 years) overlap in the morphospace, while the 3.1–6 years age group overlaps with both the two youngest and the oldest age groups, that is, 6.1–10 years. ANOVA detected significant differences between groups along PC1 (df = 3, *F* test = 33.11, *p* = <0.001), while differences along PC2 are not significative (df = 3, *F* test = 0.491, *p* = 0.69). Kruskal–Wallis highlighted the pairs of age classes that yielded significant differences on PC3 scores (chi‐squared = 10.507, df = 3, *p* value = 0.014). Tukey's post‐hoc test and Dunn's test results are shown in Tables 6 and 7. Pearson's product–moment correlation coefficient showed that only PC1 is highly correlated with size (*r* = 0.86; *p* value = <0.001), that is, with ontogenetic allometry, even though it is not completely driven by it (Figure S1).

**TABLE 6:** Results of the Tukey's post hoc test for PC1 scores

Age groups	Diff	*p* adj
1.1–3 versus 0–1	0.01959548	0.5255907
**3.1–6 versus 0–1**	0.07977212	**0.0000067**
**6.1–10 versus 0–1**	0.13144354	**0.0000000**
**3.1–6 versus 1.1–3**	0.06017664	**0.0000067**
**6.1–10 versus 1.1–3**	0.11184806	**0.0000000**
**6.1–10 versus 3.1–6**	0.05167142	**0.0022135**

*Note*: In bold the significant values.

**TABLE 7:** Pairwise comparisons using Dunn's‐test for multiple comparisons of independent samples for PC3 scores

	0.1 year	1.1–3 years	3.1–6 years
1.1–3 years	1.000	‐	‐
3.1–6 years	**0.053**	**0.054**	‐
6.1–10 years	1.000	1.000	0.157

*Note*: In bold the significant values.

For PC1 the negative scores (i.e., the youngest individuals) describe a small and more “bean‐like” talus, with a small circular depression in the neck region (Figure 3c). The trochlea is slightly shaped, without any lateral and medial rims or posterior margins. The lateral ridge is wide and well defined, while plantarly the wide and shallow sulcus tali separates the anterior and posterior calcaneal facets. Medially, the surface is homogeneous, with no medial malleolar facet developed yet. Contrary, PC1 positive scores (Figure 3c) showed a greater development of the head, which is narrower than in the youngest specimens, and more anteriorly expanded and medially rotated. A deep depression with marked rims separates the trochlea, head, and medial malleolar facet, while the neck surface greatly expands. The trochlea is slightly wedged, with well‐defined medial and lateral margins and an increased antero‐posterior convexity, while the posterior rim is still barely visible. The lateral malleolar process is almost completely developed, and the lateral ridge has reduced, due to the development of the posterior body of the talus and the head. This is visible also in the sulcus tali area, narrower and deeper due to due expansion and development of both the anterior and posterior calcaneal facets, with the former more advanced in development than the posterior one, which has a concave shape. The groove of the *flexor hallucis longus* has not developed yet. PC2 (12.1%) accounts mainly for a global antero‐posterior elongation of the overall talus, which passes from a globular shape in the youngest individual (i.e., negative scores) to a more elongated and defined shape in the oldest individuals (i.e., positive scores). PC3 (9.9%) describes subtle shape variation, such as the development of the lateral malleolar process, lateral development of the posterior calcaneal facet that increases in size and changes in orientation, and the rotation of the head along the positive axes.

Form space analysis shows that PC1 is mostly driven by size, showing an ontogenetic trajectory (“growth‐axis”), as expected. The first three PCs account for 94.47% of the total variance, 92.02% of which is explained by PC1. Pearson's product–moment correlation showed that PC1 is completely driven by size (corr = 0.98; *p* value = <0.001) (for additional information, see Figures S2 and S3).

### 3.2 Internal morphology

**FIGURE 4:**
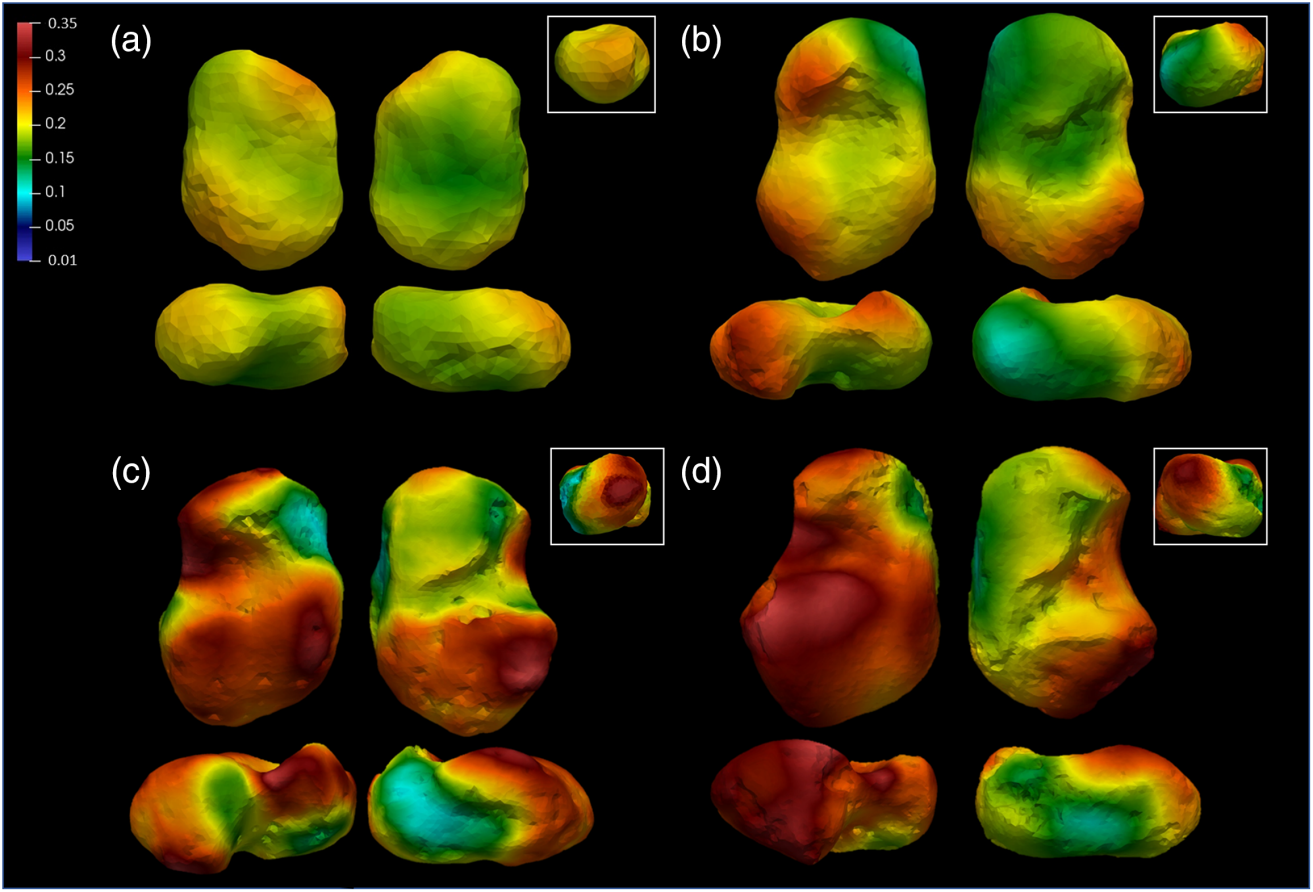
Bone volume fraction (BV/TV) group averages: (a) 0–1 year; (b) 1.1–3 years; (c) 3.1–6 years; (d) 6.1–10 years. BV/TV values are high and relatively homogenous during the first year of life; after the onset of the bipedal locomotion (>1 year), BV/TV decreases, and the magnitude is differentiated among the bone. BV/TV values start increasing again after 3 years old. The group is represented by representative specimens

**FIGURE 5:**
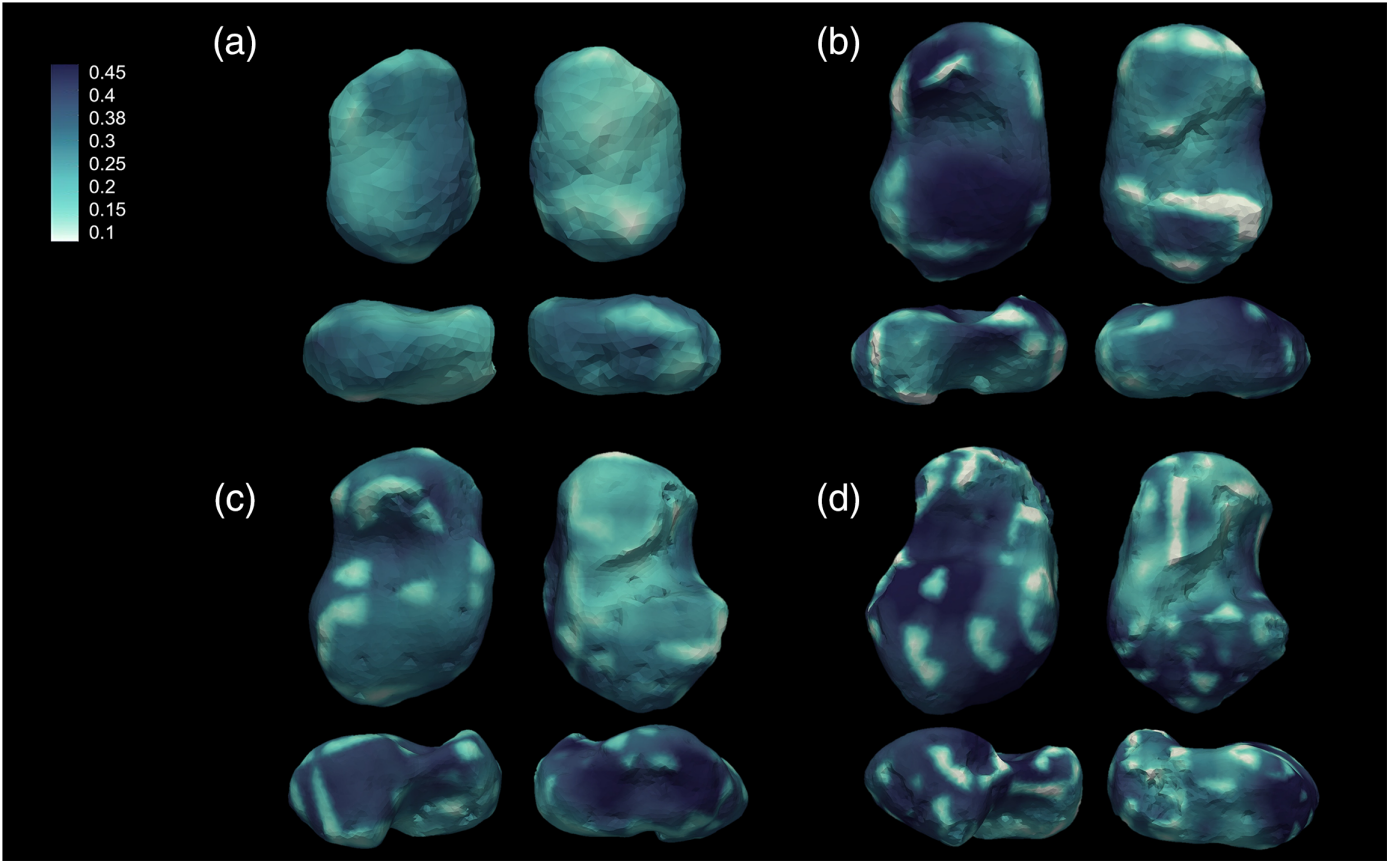
Degree of anisotropy (DA) age group averages: (a) 0–1 year; (b) 1.1–3 years; (c) 3.1–6 years; (d) 6.1–10 years. DA is low during the first year of life, that is, the talus is relatively isotropic. Anisotropy starts increasing slowly after the onset of the bipedal locomotion, at about 1 year of age, reaching the highest values after 6 years of age

**FIGURE 6:**
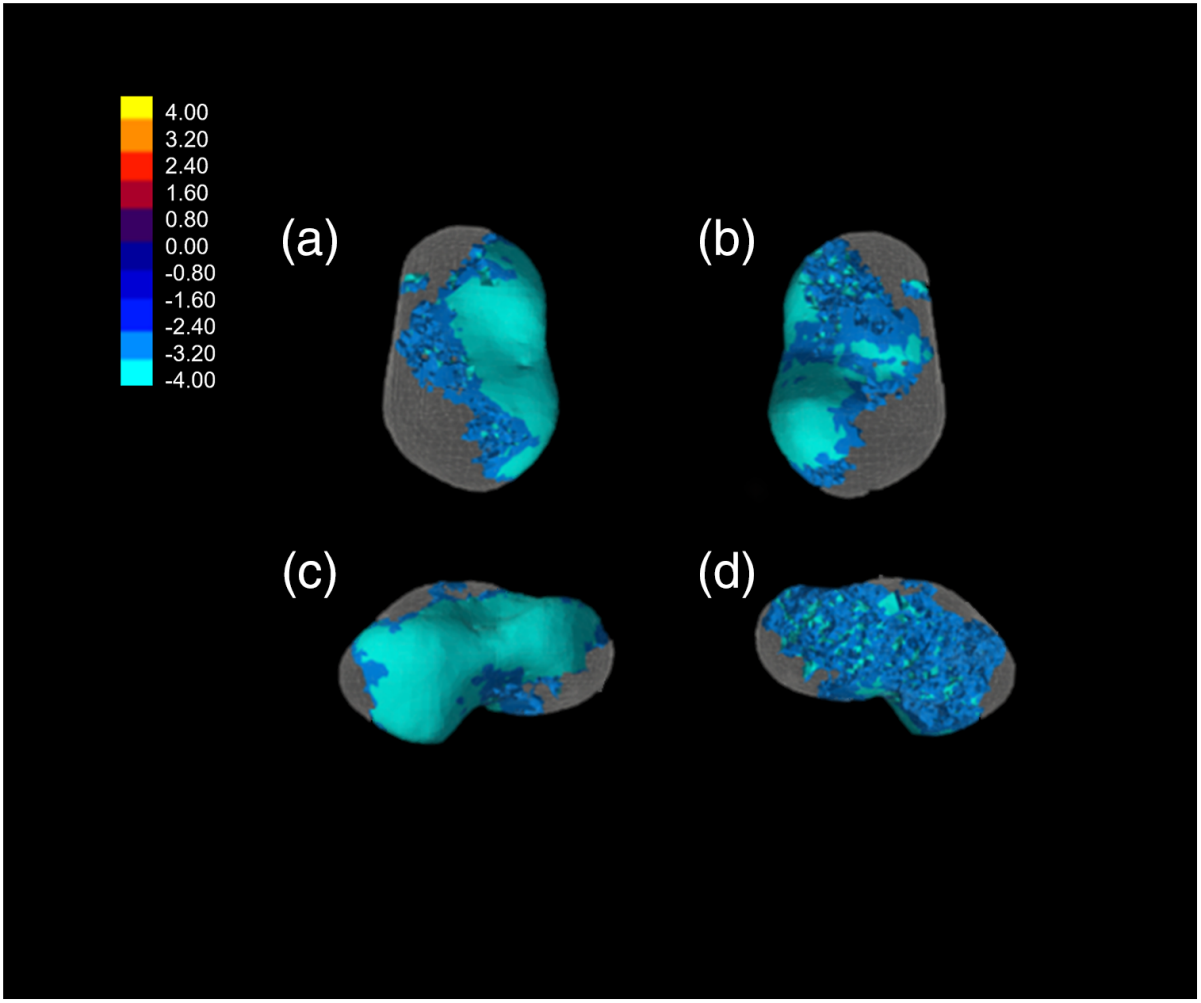
Bone volume fraction (BV/TV) T‐scores. Only the significant results are represented. Differences between the oldest age group in the sample (6.1–10 years) and 3.1–6 years show, that individuals older than 6 years of age have significantly higher values in all the lateral sides of the talus

Mean values of trabecular properties for the age classes are listed in Table 8 (Figure S6; individuals' values are listed in Tables S2–S4). During the first year, the trabecular architecture is very dense, with high BV/TV values, and numerous and thin trabeculae, very close to each other. During the first year of life, BV/TV is relatively homogeneous in the entire talus, with slightly higher values in the head and posterior part of the talar body. During the second and third years (age group 1.1–3 years, Figure 4b), BV/TV values are generally lower than during the first year, but with relatively higher values in the posterior part of the body, that is, trochlea, lateral malleolar process, lateral part of the head. Values increase again in the 3.1–6 years age group. The pattern is similar to the one described for the 1.1–3 years cohort, but with differences in magnitude, with higher values in the trochlea and posterior calcaneal facet, head, and lateral malleolar process, but with slightly lower values than the ones registered in the youngest group in the medial side of the head. In the oldest group, BV/TV pattern is once again similar to the latter description, but with differences in magnitude, that is, the highest values (Figure 4d). DA values increase after the first year of life. At about 1.5 years trochlea, neck, dorsal part of the head, and posterior subtalar facet show the highest DA values. After 3 years of age, DA increases also in the medial malleolar facet area, while at about 6 years, higher values can be noticed in the most distal part of the head, that is, navicular facet, posterior subtalar joint, and lateral malleolar process. This pattern remains constant after 7–8 years (Figure 5d). *T* test results are shown in Figure 6. The BV/TV and DA ranges within each age group are shown in Figures S4 and S5.

**TABLE 8:** Mean values and standard deviation for age classes

Age class	DA (SD)	BV/TV (SD)	Tb.N (SD)	Tb.Sp (SD)	Tb.Th (SD)
0–1 year	0.18 (0.04)	17.71 (0.05)	1.54 (0.36)	0.54 (0.15)	0.14 (0.03)
1.1–3 years	0.22 (0.04)	15.99 (0.04)	1.15 (0.13)	0.70 (0.10)	0.19 (0.03)
3.1–6 years	0.22 (0.03)	17.51 (0.03)	1.20 (0.16)	0.65 (0.09)	0.20 (0.03)
6.1–10 years	0.22 (0.03)	22.83 (0.06)	1.15 (0.11)	0.65 (0.07)	0.23 (0.05)

## 4 DISCUSSION

This work investigates the talar internal and external morphologies during postnatal growth up to about 10 years of age. We found that both morphologies differ significantly at different stages, potentially reflecting the variable joint positions and load distribution during the development of bipedal locomotion. These results are consistent with previous ontogenetic studies of other skeletal elements (Colombo et al., 2019; Saers et al., 2020) and the talus (Figus et al., 2022; Hellier & Jeffery, 2006).

### 4.1 External morphology

In shape space, each age group differs significantly from each other in terms of PC1 scores, with the sole exception of the first two classes, 0–1 and 1.1–3 years, while the former significantly differs from age group 3.1–6 in PC3 scores, which is expected considering the age gap. The differences in PC1 scores may be justified not only by changes in shape but also by ontogenetic allometry, that is, morphological variation strictly correlated with an increase in size. Form space showed significant differences among all age classes in terms of PC1 scores, which is completely driven by size increase during growth, especially during the first years. At birth, the foot has reached about 1/3 of its final length (Dimeglio & Stanitski, 2001; Fritz & Mauch, 2013; Maier & Killmann, 2003) and, during the first 3 years of life, it achieves about 2/3 of the final length (Fritz & Mauch, 2013; Maier & Killmann, 2003; Volpon, 1994). This rapid development is needed to bear the increasing body weight and makes the foot the first body segment to complete the growth process (Dimeglio & Stanitski, 2001).

Neonates and infants under 1 year show a less well‐defined talus, with a round and shallow neck area, a rounded head, and a small body, with a very short trochlea. The medial and lateral malleolar facets are not developed yet, while the anterior and posterior calcaneal facets are already distinguishable. This morphology likely reflects a scarce experience in locomotion (Chagas et al., 2006), as the balance and movement coordination skills are still developing (Adolph et al., 2003; Clark et al., 1988; Sutherland et al., 1980; Thelen, 1992). During this phase, the infant engages in non‐loading movements. They start to crawl and try to stay fully erect only in the second half of the first year when the flat‐foot completely touches the ground to increase their balance (Sutherland et al., 1980).

By age one, toddlers (1.1–3) show advancement in the development of the lateral malleolar process, and the head commences its medial rotation, while the neck starts to lengthen. It is important to highlight that, during this period, the talus is not completely ossified and is still surrounded by a more elastic cartilaginous tissue, potentially absorbing some forces. At the same time, most of the developmental locomotor milestones take place. The higher mediolateral trunk oscillations, and the consequent variation in the ankle joint angles, may be correlated to the small development of the malleolar processes, also probably influenced by the neutralization of the valgus inclination of the ankle. The anterior calcaneal facet develops slightly faster than the posterior one, which is triangle‐shaped. The highly unbalanced and variable form of locomotion that infants adopt during this phase, with the foot completely adhering to the ground, may explain the slow development of the calcaneal facets and, in particular, the posterior one. This is probably because a proper heel strike only reportedly develops after 18–24 months (Zeininger, 2013). At this stage, the foot is still touching the ground completely with an immature roll‐off. The medial areas of the foot greatly contribute to the bearing of the load, probably connected to the initial and slight change in the head orientation. The toddlers's foot is characterized by a flat arch profile with large contact areas and a lot of subcutaneous fat. This condition changes with the development of the longitudinal arch, which reaches the adult shape between 5 and 6 years of age (Bertsch et al., 2004). This process is driven by genetic and epigenetic factors, such as body weight, physical activity, and footwear (Fritz & Mauch, 2013). There is a general consensus on the link between the longitudinal arch development and the onset of bipedalism. In addition, the development of the lateral and medial malleolar facets changes the aspect of the trochlea, determining an increase in the lateral and medial rims and a deeper concavity, potentially correlated with the rotation of the malleoli of the ankle. This phase is completed around 5–6 years (Fritz & Mauch, 2013), with a subsequent increase in stability of the ankle joint. In addition, the enlargement of the trochlear surface makes it more efficient in receiving the body weight from the tibia (Pal & Routal, 1998). All these considerable changes in the mechanics of gait may explain this difference found in our sample.

After age three (Early childhood, 3.1–6 years) an elongation of the overall talus and trochlea is manifest, which is slightly centrally wedged, with more evident medial and lateral rims. The head continues to rotate medially and the lateral malleolar process proceeds to develop assuming an inferior and lateral projection. As the talus grows in a dorsal‐palmar direction, the sulcus tali becomes deeper. The anterior and posterior calcaneal facets continue to develop. The former points ahead while the latter starts to develop its characteristic concavity and appears more rectangular, especially after age six. During this time, a slight rotation of the trochlea is also visible and becomes more evident after 6 years of age. All these characteristics may likely reflect a more mature gait pattern. The conspicuous change in talar shape that occurs at about 3 years is denoted by the slight development of the lateral malleolar process, an increased trochlear curvature, and medial rotation of the head. This modification may correspond to a particular milestone, that is, when the heel‐strike pattern has developed, and an adult‐like transfer of the forces from the lateral to the medial side makes its appearance. Furthermore, the development of the medial longitudinal arch may account for the modeling of the talar head and neck. The patterns of pressure distribution change from a more uniformly distributed load to a more skewed, adult‐like pattern, where the COP is concentrated under the heel at heel‐strike. Then it moves laterally and to the front, finally shifting medially in preparation for toe‐off.

During late childhood (6.1–10 years), children present with a generally more mature morphology, with more defined lateral and medial malleolar facets. The medial malleolus begins ossifying at about 3–5 years and completes its ossification at about 8 years of age (Turley & Frost, 2014b). Only the posterior part of the body, for example, the posterior rim of the trochlea and the posterior subtalar facets, has not yet reached the adult morphology. In this phase, the head and neck complete their medial rotation and appear more plantarly oriented. This feature is commonly associated with the presence of the medial longitudinal arch (Day & Wood, 1968; Gebo, 1992; Prang, 2015; Sorrentino, Carlson, et al., 2020). This feature greatly increases between 5 and 6 years (Bertsch et al., 2004), as reflected by our results. We show that a more medially rotated head and declined neck appear during the toddling phase (3.1–6) and continue their development during childhood (6.1–10).

Finally, we observed an increase in the size of the articular facets and orientation of the head, trochlea, and posterior calcaneal facet, with consequent reduction of the non‐articular areas, for example, the sulcus tali and neck, ultimately confirming our initial hypothesis. Hellier and Jeffery (2006) described the plasticity of juvenile talus on a sample of juvenile tali of individuals aged between 8 and 18 years, arguing that the articular facets respond to an increase in loading by altering their shape and orientation, causing a directional change in the forces passing through them. We observed that this coping phenomenon is present also before this age range. Between three and 6 years, the trochlea and head start to modify their orientation. From age 6, the posterior subtalar facet also starts to rotate toward a more medio‐plantar direction. This is probably linked to the fact that the trochlea receives compressive forces from the tibia, and they pass directly to the posterior calcaneal facet, downwards and forwards, to the neck (Hellier & Jeffery, 2006). Here the compressive forces are converted into tensile forces and change direction to pass to the head, from where they are transmitted to the navicular and the calcaneus. Furthermore, when standing, the forces are not distributed homogeneously, as a higher amount of them are transmitted medially to the metatarsal, particularly to the hallux (Hellier & Jeffery, 2006).

### 4.2 Internal morphology

Trabecular results show that the architecture of the talus changes through ontogeny and accompanies the development of human bipedal locomotion. All age classes show a trend in which mean BV/TV and Tb.N start with high values, while Tb.Th and Tb.Sp exhibit low values. After 1 year of age, the trend is inverted: BV/TV and Tb.N values start decreasing, while Tb.Th and Tb.Sp increase progressively until 10 years of age, supporting our initial hypotheses. In our study, statistical differences are present in BV/TV and DA values among age classes. BV/TV differs significantly between the 3.1–6 and 6.1–10 groups in the lateral side of the talus, sulcus tali, and lateral side of the head. From being a dense and non‐specialized structure with a high number of thin and closely spaced struts and an isotropic structure, the talar architecture becomes a highly adaptive, anisotropic structure, with less dense and thicker trabeculae. This result is in line with the literature and supports our initial hypotheses. Excessive bone laid out during the gestational period represents a fundamental calcium reservoir (Acquaah et al., 2015) in the postnatal period, when the reduced quantities in breast milk may not be sufficient for the infant's needs (Ilich & Kerstetter, 2013). On the other hand, it becomes soon excessive and is consequently reabsorbed. The high bone density around birth may reflect a gestational overproduction (Acquaah et al., 2015; Milovanovic et al., 2017). Denser bone in neonates may be the result of the endochondral ossification when the bone is quickly laid out following a genetic blueprint and the rapid growth of the cartilage anlagen (Milovanovic et al., 2017). Subsequent (re)modeling may also be genetically driven and the result of adaptation to muscle contractions generated by bipedal gait, as confirmed by our results. After age one, the trabeculae are more anisotropic and thicker, and BV/TV stops decreasing after the onset of unassisted locomotion, as a response to the load. After the third year of age, BV/TV increases once again as a consequence of weight gain and increasing compressive and tensile forces that pass through the talus. The peak is shown in the oldest age cohort, that is, 6.1–10 years. The highest values are found in the posterior and lateral sides of the talar body, possibly due to an increased load on the hindfoot following heel strike. Overall, after the first year, trabecular architecture is affected by increased and differential loading, as suggested by previous works on other skeletal elements conducted with different methodologies (Colombo et al., 2019; Gosman & Ketcham, 2009; Milovanovic et al., 2017; Raichlen et al., 2015; Ryan et al., 2017; Ryan & Krovitz, 2006; Saers et al., 2020).

DA values differ in magnitude between the youngest group and all the others, especially in the medial side and head, the lateral and medial part of the trochlea, the dorsal trochlear surface, and the lateral process. During the first year, the trabecular architecture is isotropic and shows a consistent increase after this period, that is, at the onset of locomotion, marking the rise in loading. This pattern is consistent with an immature gait and high mediolateral trunk oscillations which are reflected also in the ankle joint, as well as with the absence of a stereotyped “heel‐to‐toe” roll‐over pattern and the characteristic foot dorsiflexion. This pattern is different from what Ryan and Krovitz (2006) found in the proximal femur, as in one of the two studied VOIs, the initial structure was anisotropic, highlighting a probable genetic blueprint (Cunningham & Black, 2009a, 2009b), but is in line with previous works (Colombo et al., 2019; Figus et al., 2022; Saers et al., 2020). DA then increases steadily until the third year of age, as the gait pattern becomes more mature and stereotyped and adult‐like, that is, when the ankle joint is more stable. Some changes occur locally, and bone portions that are most affected by the increased loading show thicker trabeculae and higher DA. The most significant variations are observed around 7–8 years, before the development of a mature gait. Areas presenting with the highest values are the trochlea, which is pivotal in receiving and distributing the forces in a postero‐anterior way when the tibia rolls over the trochlear surface, and the head. The latter probably converts the compressive forces to tensile forces in the neck, finally transmitting them forward toward the navicular and the medial side of the foot. DA also increases on the medial and lateral sides, probably reflecting a shift in loading toward the lateral side of the foot. As seen above, the development of the lateral and medial sides of the trochlea is correlated with the development and rotation of the malleoli (Fritz & Mauch, 2013), which helps the ankle gain stability during standing and walking around 5–6 years. The plantar surface shows a late increase in DA values, especially after 6 years of age, probably linked to the higher peak plantar pressures on the fore‐ and hind‐foot.

### 4.3 Are changes in internal and external structure a marker for locomotor milestones?

Overall, our results show that the most interesting morphological changes are observed before the age of six, when the most important changes in articular facet orientation and BV/TV also take place. Talar shape, however, exhibits a slower development.

During the first year of life, especially during the first 6 months, both external and internal talar morphologies are not “specialized.” When looking at the external shell, the globular and almost facet‐free talus tells us that it is not yet ready to sustain its role of receiver and distributor of forces. And the trabecular architecture confirms it, showing a correspondingly unspecialized architecture, with a dense and isotropic structure that does not yet display any evidence of real loading. Between the end of the first year and after the beginning of the second year toddlers usually start to walk engaging in initially unstable locomotion, which involves the absence of a heel strike and toe‐off and a flat foot that touches the ground. Talar shape reflects these improvements, with slight changes in the medial orientation of the head, trochlear surface, lateral malleolar process, and posterior calcaneal facet. Consequently, the trabecular architecture, especially in the anterior calcaneal facet, records the variation in strain with a decrease in BV/TV and fewer, thicker, and more widely spaced trabeculae. The non‐loaded trabeculae are resorbed, promoting the modeling of oriented struts. In age class 3.1–6, talar shape shows an increase in trochlear concavity, development of the facets, and an overall bone elongation. Trabecular bone tracks these changes, and BV/TV starts to increase again in the same areas as the ones in which the greatest increase in size and orientation is recorded: trochlea, subtalar posterior facet, lateral malleolar process, and the most distal portion of the head. At the same time, DA increases, with higher values in the trochlea and head. These transformations are potentially linked to changes in gait pattern and foot function with the development of propulsive toe‐off and heel‐strike, while the longitudinal arch is still developing. Finally, at 6.1–10 years, when the gait reaches an adult‐like pattern, talar morphology shows variations that may be related to the transfer of weight from the lateral side to the medial side in preparation for the push‐off, as well as to the development of the medial longitudinal arch. The same areas are affected by an increase in BV/TV magnitude. DA continues to increase in the most dorsal parts of the trochlea, neck, head, lateral and medial malleolar facets, and posterior calcaneal facets. The remarkable changes observed in this study may reflect changes in gait patterns, contributing to tracking the main locomotor milestones.

## 5 CONCLUSION

The results of this work add to a growing literature supporting the pivotal role that mechanical loading plays in modeling the cortical and trabecular bones during growth (Barak et al., 2011; Raichlen et al., 2015; Ryan et al., 2017). The information obtained from this work points out how important a holistic approach is when investigating the ontogenetic changes linked to the development of bipedal locomotion. Our research shows that the plasticity of human talus, even though driven by a genetic blueprint, may display an epigenetic influence. Moreover, when accompanied by the study of trabecular bone, it may open the doors to a whole new investigation strategy. However, a few drawbacks that may affect the work are to be taken into account. In this study, the samples vary geographically, culturally, and chronologically. We do not know to what extent the genetic and cultural differences may act on the talus. Another issue could be using an archeological sample, with the sole exception of the individuals from Bologna. In this case, we did not know whether the causes of death affected their behavior or their locomotor development. Additionally, the sample is too small to permit the analysis on a year‐by‐year basis, even if this would be helpful with more balanced specimens. Moreover, the trabecular statistical results are to be taken with caution because of the enormous morphological changes to which the talus is subject during growth. It should be considered only as an additional visual instrument in the description of the morphological variations. Finally, our approach, that is, combining the external and internal investigation of the talus in an ontogenetic framework, may help in shedding light on the long search for the origin of human bipedalism, most importantly, when the study of fossils is concerned.

## AUTHOR CONTRIBUTIONS

**Carla Figus:** Conceptualization‐Lead, Data curation‐Lead, Formal analysis‐Equal, Investigation‐Lead, Validation‐Equal, Visualization‐Equal, Writing – original draft‐Lead, Writing – review & editing‐Equal. **Nicholas B. Stephens:** Data curation (supporting); formal analysis (equal); software (lead); supervision (supporting); validation (equal); visualization (equal); writing – review and editing (supporting). **Rita Sorrentino:** Data curation (supporting); formal analysis (supporting); writing – review and editing (supporting). **Eugenio Bortolini:** Formal analysis (supporting); validation (supporting); writing – review and editing (supporting). **Simona Arrighi:** Writing – review and editing (supporting). **Federico Lugli:** Writing – review and editing (supporting). **Giulia Marciani:** Writing – review and editing (supporting). **Gregorio Oxilia:** Writing – review and editing (supporting). **Matteo Romandini:** Writing – review and editing (supporting). **Sara Silvestrini:** Writing – review and editing (supporting). **Fabio Baruffaldi:** Resources (supporting); writing – review and editing (supporting). **Maria Giovanna Belcastro:** Resources (supporting); writing – review and editing (supporting). **Federico Bernardini:** Resources (supporting); writing – review and editing (supporting). **Igor Erjavec:** Resources (supporting); writing – review and editing (supporting). **Anna Festa:** Resources (supporting). **Tamás Hajdu:** Resources (supporting); writing – review and editing (supporting). **Orsolya Mateovics‐László:** Resources (supporting); writing – review and editing (supporting). **Mario Novak:** Resources (supporting); writing – review and editing (supporting). **Ildikó Pap:** Resources (supporting); writing – review and editing (supporting). **Tamás Szeniczey:** Writing – review and editing (supporting). **Claudio Tuniz:** Resources (supporting); writing – review and editing (supporting). **Timothy M. Ryan:** Conceptualization (supporting); resources (supporting); supervision (equal); validation (equal); writing – review and editing (supporting). **Stefano Benazzi:** Conceptualization (supporting); funding acquisition (lead); project administration (lead); resources (lead); supervision (equal); validation (equal); writing – review and editing (supporting).

## Supporting information


**FIGURE S1** Correlation with size in shape space (PC1 vs logCn)
**FIGURE S2** 3D form space plot
**FIGURE S3** Correlation between PC1 and centroid size
**FIGURE S4** BV/TV ranges within each age group.
**FIGURE S5** DA ranges within each age group.
**FIGURE S6** Mean values of trabecular properties are represented for each age group.
**TABLE S1.** Study sample. The specimens are ordered by age, from the youngest to the oldest
**TABLE S2** Individuals' averages for BV/TV and DA
**TABLE S3** Individuals' averages for Tb.N, Tb.Sp and Tb.Th

## Data Availability

The data that support the findings of this study are available from the corresponding author upon reasonable request.
