# Supplementary material for: Human talar ontogeny: Insights from morphological and trabecular changes during postnatal growth
Source: Am J Biol Anthropol. 2022 Aug 6;179(2):211–28. doi: 10.1002/ajpa.24596 (PMC9804293; doi:10.1002/ajpa.24596)
Supplement: Supplementary file 1 — FIGURE S1 Correlation with size in shape space (PC1 vs logCn) FIGURE S2 3D form space plot FIGURE S3 Correlation between PC1 and centroid size FIGURE S4 BV/TV ranges within each age group. FIGURE S5 DA ranges within each age group. FIGURE S6 Mean values of trabecular properties are represented for each age group. TABLE S1. Study sample. The specimens are ordered by age, from the youngest to the oldest TABLE S2 Individuals' averages for BV/TV and DA TABLE S3 Individuals' averages for Tb.N, Tb.Sp and Tb.Th [file AJPA-179-211-s001.docx]

**Supplementary Information**

**Human talar ontogeny: insights from morphological and trabecular changes during postnatal growth**

Carla Figus, Nicholas B Stephens, Rita Sorrentino, Eugenio Bortolini, Simona Arrighi, Federico Lugli, Giulia Marciani, Gregorio Oxilia, Matteo Romandini, Sara Silvestrini, Fabio Baruffaldi, Maria Giovanna Belcastro, Federico Bernardini, Igor Erjavec, Anna Festa, Tamas Hajdu, Orsolya Mateovics-László**,** Mario Novak, Ildiko Pap, Tamás Szeniczey, Claudio Tuniz, Timothy M Ryan, Stefano Benazzi

Table S1. Study sample. The specimens are ordered by age, from the youngest to the oldest

| **Site** | **Specimen** | **Sex** | **Period** | **Age at death** | **Age class** | **Cause of death** |
| --- | --- | --- | --- | --- | --- | --- |
| **Norris Farm** | 821369 |  | 1300 CE | 8 weeks | 0-1 years |  |
| **Velia** | T305 us1344 |  | ~100-200 CE | 0-3 months |  |  |
| **Norris Farm** | 821045 |  | 1300 CE | 3 months |  |  |
| **Velia** | T300 us1167 |  | ~100-200 CE | 0-6 months |  |  |
| **Velia** | T441 us2538 |  | ~100-200 CE | 0-6 months |  |  |
| **Velia** | T368 us2069 |  | ~100-200 CE | 0.75-1 year |  |  |
| **Velia** | T398 us2239 |  | ~100-200 CE | 6-8 months |  |  |
| **Velia** | T442 us2545 |  | ~100-200 CE | 6-9 months |  |  |
| **Norris Farm** | 821051 |  | 1300 AD | 7.5 months |  |  |
| **Bologna** | **BO58** | **M** | **20^th^ Century** | **11 months** |  | **Bronchitis** |
| **Bologna** | **BO60** | **F** | **20^th^ Century** | **11 months** |  | **Acute meningitis (brain fever)** |
| **Velia** | T289 us1098 |  | ~100-200 CE | 9-12 months |  |  |
| **Velia** | T434 us2454 |  | ~100-200 CE | 1-1.5 years | 1.1-3 years |  |
| **Velia** | T415 us2344 |  | ~100-200 CE | 1-1.5 years |  |  |
| **Norris Farm** | 821046 |  | 1300 CE | 1.5 years |  |  |
| **Norris Farm** | 821014 |  | 1300 CE | 1.5 years |  |  |
| **Bologna** | **BO14** | **M** | **20^th^ Century** | **1 years 5 months** |  | **Chronic enteritis** |
| **Perkáta-Nyúli dűlő** | 516 |  | 14-16th c. | 1.5-3 years |  |  |
| **Perkáta-Nyúli dűlő** | 655 |  | 14-16th c. | 1-3 years |  |  |
| **Ilok-Kraljevića Street** | G24 |  | 16^th^-17^th^ Century | 1.5-2 years |  |  |
| **Velia** | T286 us1071 |  | ~100-200 CE | 1.5-2 years |  |  |
| **Bologna** | **BO14** | **F** | **20^th^ Century** | **1 year 9 months** |  | **Enteritis** |
| **Norris Farm** | 821026 |  | 1300 CE | 2 years |  |  |
| **Norris Farm** | 821207 |  | 1300 CE | 2 years |  |  |
| **Norris Farm** | 821069 |  | 1300 CE | 2.5 years |  |  |
| **Norris Farm** | 821113 |  | 1300 CE | 2.5 years |  |  |
| **Perkáta-Nyúli dűlő** | 639 |  | 14^th^-16^th^ Century. | 2.5-3 years |  |  |
| **Perkáta-Nyúli dűlő** | 3421 |  | 10^th^-12^th^ Century. | 2.5-3.5 years |  |  |
| **Ilok-Krstbajer** | G22 |  | 13^th^-15^th^ Century | 2-3 years |  |  |
| **Velia** | T379 us2143 |  | ~100-200 CE | 2-3 years |  |  |
| **Velia** | T411 us2319 |  | ~100-200 CE | 2-3 years |  |  |
| **Bologna** | **BO7** | **M** | **20^th^ Century** | **2 years 9 months** |  | **Intestinal gastroenteritis** |
| **BeliManastir** | G6 |  | ~4800-4500 BCE | 2-3.5 years |  |  |
| **Norris Farm** | 820683 |  | 1300 CE | 3 years |  |  |
| **Norris Farm** | 819938 |  | 1300 CE | 3 years |  |  |
| **Norris Farm** | 821214 |  | 1300 CE | 3 years |  |  |
| **Bologna** | **BO48** | **F** | **20^th^ Century** | **3 years** |  | **Meningitis** |
| **Ilok-Krstbajer** | G72 |  | 13^th^-15^th^ Century | 3-4 years | 3.1-6 years |  |
| **Ilok-Krstbajer** | G70 |  | 13^th^-15^th^ Century | 3-4 years |  |  |
| **Ilok-Krstbajer** | G1 |  | 13^th^-15^th^ Century | 3.5-4.5 years |  |  |
| **Paks TO-18** | 997 |  | 14^th^-16^th^ Century. | 3-5 years |  |  |
| **Paks TO-18** | 1166 |  | 14^th^-16^th^ Century. | 3-5 years |  |  |
| **Velia** | T209 us1379 |  | ~100-200 CE | 4 years ca |  |  |
| **Velia** | T342 us1546 |  | ~100-200 CE | 4 years ca |  |  |
| **Paks TO-18** | 1846 |  | 14^th^-16^th^ Century. | 4-5 years |  |  |
| **Perkáta-Nyúli dűlő** | 4263 |  | 10^th^-12^th^ Century. | 4-5 years |  |  |
| **Norris Farm** | 821012 |  | 1300 CE | 5 years |  |  |
| **Velia** | T375 us2129 |  | ~100-200 CE | 5 years |  |  |
| **Bologna** | **BO5** | **F** | **20^th^ Century** | **5 years** |  | **Acute meningitis** |
| **Bologna** | **Parma7** | **F** | **20^th^ Century** | **5 years** |  | **Diphteria** |
| **Bologna** | **BO6** | **F** | **20^th^ Century** | **5 years 10 months** |  | **Enteroperitonitis** |
| **Bologna** | **BO1** | **M** | **20^th^ Century** | **5 years 8 months** |  | **Acute enteritis** |
| **BeliManastir** | G20 |  | ~4800-4500 BCE | 5.5-6.5 years |  |  |
| **Paks TO-18** | 1164 |  | 14^th^-16^th^ Century. | 5-6 years |  |  |
| **Ilok-Krstbajer** | G55 |  | 13^th^-15^th^ Century | 5-6 years |  |  |
| **Velia** | T390 us2207 |  | ~100-200 CE | 5-6 years |  |  |
| **Bologna** | **BO4** | **F** | **20^th^ Century** | **5 years** |  | **Bronchopneumonia, basilar meningitis** |
| **Bologna** | **BO11** | **F** | **20^th^ Century** | **6 years** |  | **Typhus** |
| **Perkáta-Nyúli dűlő** | 435 |  | 14^th^-16^th^ Century. | 6-7 years | 6.1-10 years |  |
| **Velia** | T333 us1489 |  | ~100-200 CE | 6-7 years |  |  |
| **Perkáta-Nyúli dűlő** | 1575 |  | 10^th^-12^th^ Century. | 6-8 years |  |  |
| **Bologna** | **BO6** | **M** | **20^th^ Century** | **7 years** |  | **Abdominal typhus, peritoneal with perforation** |
| **Paks TO-18** | 1865 |  | 14^th^-16^th^ Century. | 7-8 years |  |  |
| **Perkáta-Nyúli dűlő** | 752 |  | 14^th^-16^th^ Century. | 7-8 years |  |  |
| **Perkáta-Nyúli dűlő** | 2123 |  | 10^th^-12^th^ Century | 7-8 years |  |  |
| **BeliManastir** | G31 |  | Middle Neolithic | 7-8 years |  |  |
| **Velia** | T320 us1431 |  | ~100-200 CE | 7-8 years |  |  |
| **BeliManastir** | G1 |  | ~4800-4500 BCE | 7-9 years |  |  |
| **Bologna** | **BO40** | **M** | **20^th^ Century** | **9 years** |  | **Congenital myxedema** |
| **Velia** | T138 us1636 |  | ~100-200 CE | 9-10 years |  |  |


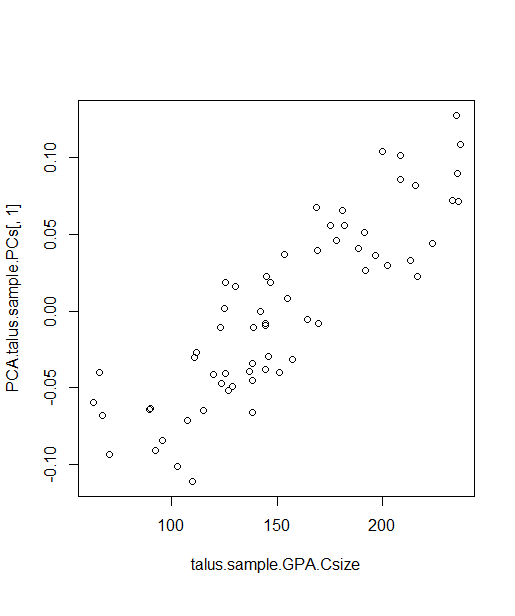


Figure S1 - Correlation with size in shape space (PC1 vs logCn)

#


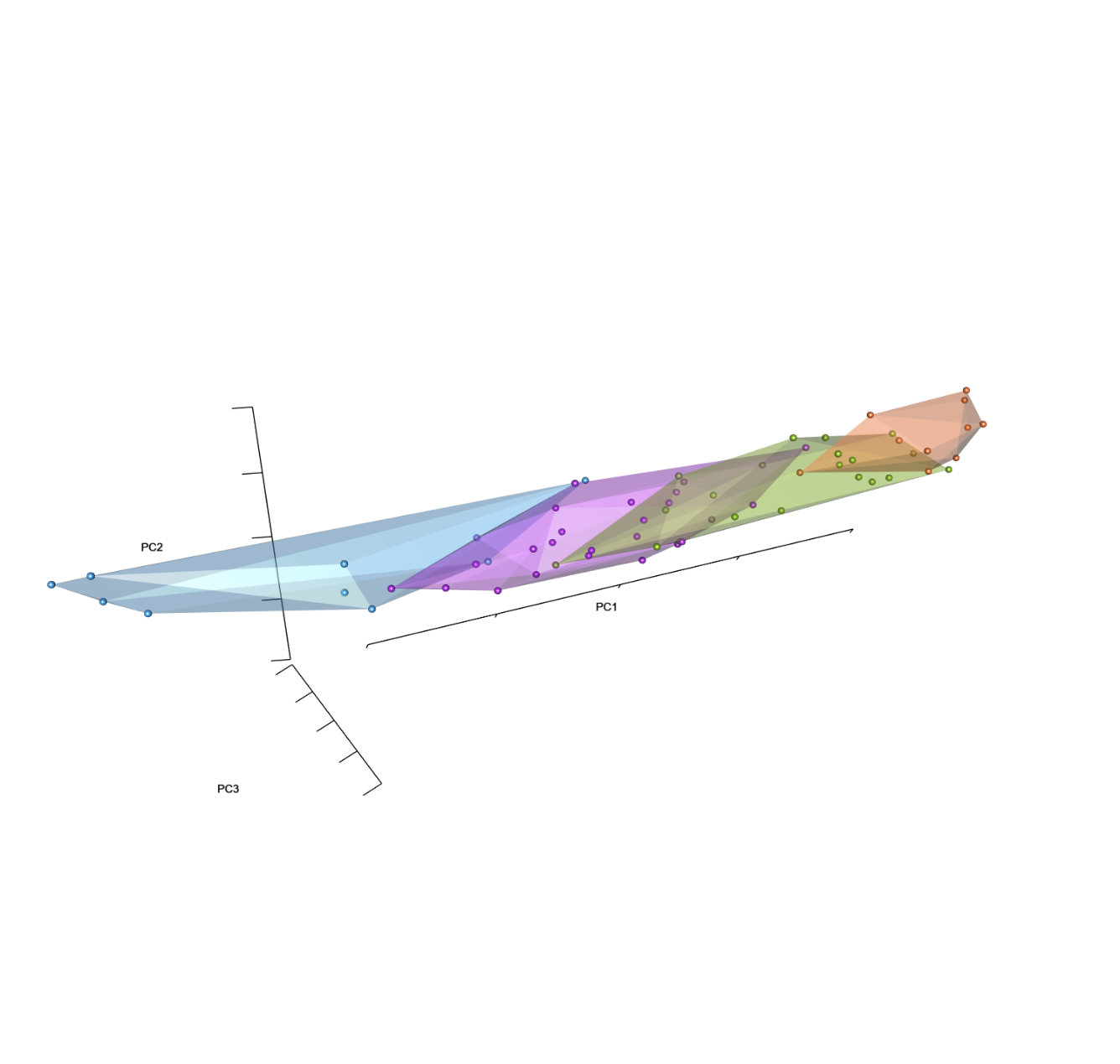

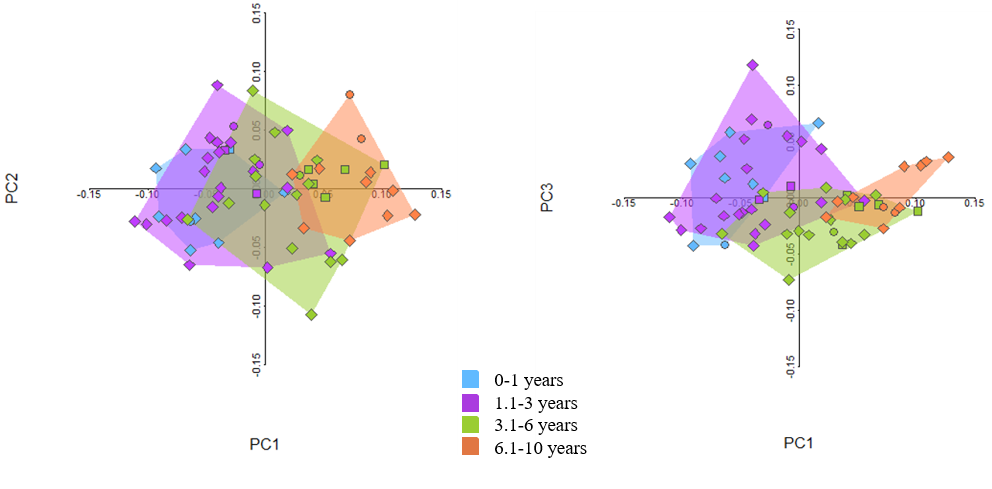


Figure S2 - 3D form space plot


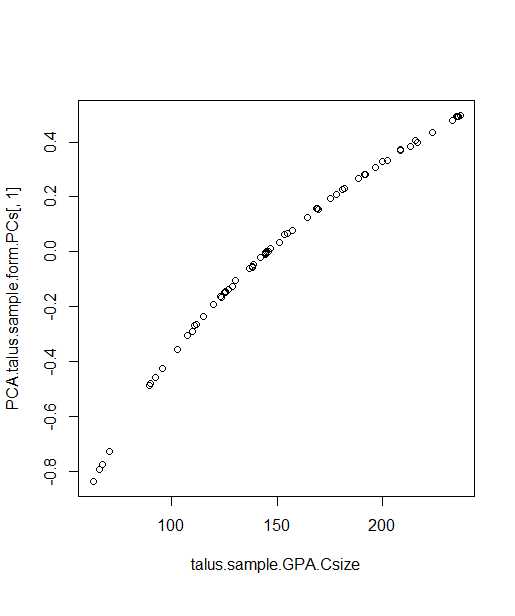


Figure S3 - Correlation between PC1 and centroid size

Table S2 – Individuals’ averages for BV/TV and DA

| Site | Specimen | Age Class | Age at death | BV/TV (%) | DA |
| --- | --- | --- | --- | --- | --- |
| Velia | T305. | Neonates and toddlers | 0-3 months | 21.65 | 0.16 |
| Norris Farms | 821369. |  | 8 weeks | 9.82 | 0.12 |
| Norris Farms | 821045. |  | 0-6 months | 23.68 | 0.11 |
| Velia | T300. |  | 0-6 months | 12.41 | 0.26 |
| Velia | T441. |  | 0-6 months | 21.92 | 0.17 |
| Norris Farms | 821051. |  | 7.5 months | 18.53 | 0.16 |
| Velia | T442. |  | 6-9 months | 18.12 | 0.19 |
| Velia | T368. |  | 0.75-1 year | 18.95 | 0.17 |
| Velia | T289. |  | 9-12 months | 18.47 | 0.16 |
| Bologna | 58_M. |  | 11 months | 23.24 | 0.16 |
| Bologna | 60_F. |  | 11 months | 20.12 | 0.26 |
| Norris Farms | 820614. |  | 12 months | 5.7 | 0.24 |
| Velia | T415. | Early Infancy | 1-1.5 years | 20.66 | 0.13 |
| Velia | T434. |  | 1-1.5 years | 18.6 | 0.27 |
| Bologna | 14_M. |  | 1year and 5 months | 11.8 | 0.23 |
| Norris Farms | 821014. |  | 1.5 years | 28.42 | 0.17 |
| Norris Farms | 821046. |  | 1.5 years | 14.84 | 0.20 |
| PerkataNyuli | 655. |  | 1-3 years | 10.93 | 0.24 |
| Velia | T286. |  | 1.5-2 years | 21.31 | 0.16 |
| Bologna | 14_F. |  | 1.9 years | 13.82 | 0.26 |
| Bologna | 48_F. |  | 3 years | 15.247 | 0.25 |
| Norris Farms | 820683. |  | 3 years | 19.14 | 0.26 |
| Norris Farms | 821026. |  | 2 years | 10.58 | 0.20 |
| Norris Farms | 821207. |  | 2 years | 10.51 | 0.21 |
| Norris Farms | 821214. |  | 3 years | 8.71 | 0.25 |
| PerkataNyuli | 516. |  | 1.5-3 years | 17.18 | 0.21 |
| Ilok | G22. |  | 2-3 years | 10.5 | 0.19 |
| Norris Farms | 821069. |  | 2.5 years | 19.6 | 0.27 |
| Norris Farms | 821113. |  | 2.5 years | 12.76 | 0.26 |
| Velia | T411. |  | 2-3 years | 18.39 | 0.20 |
| Beli Manastir | G6. |  | 2-3.5 years | 21.12 | 0.17 |
| PerkataNyuli | 639. |  | 2.5-3 | 13.98 | 0.26 |
| Bologna | 7_M. |  | 2.9 years | 16.3683 | 0.25 |
| PerkataNyuli | 3421. |  | 2.5-3.5 years | 17.48 | 0.24 |
| Norris Farms | 819938. | Late Infancy | 3 years | 12.27 | 0.25 |
| Ilok | G70. |  | 3-4 years years | 14.09 | 0.16 |
| Ilok | G72. |  | 3-4 years | 15.39 | 0.16 |
| Paks | 1166. |  | 3-5 years | 27.02 | 0.17 |
| Paks | 997. |  | 3-5 years | 16.58 | 0.25 |
| Velia | T209. |  | 4 years | 13.85 | 0.30 |
| Velia | T342. |  | 4 years | 15.82 | 0.22 |
| Paks | 1846. |  | 4-5 years | 20.88 | 0.20 |
| PerkataNyuli | 4263. |  | 4-5 years | 21.43 | 0.20 |
| Bologna | 4_F. |  | 5 years | 18.5 | 0.21 |
| Bologna | 5_F. |  | 5 years | 16.22 | 0.22 |
| Norris Farms | 821012. |  | 5 years | 10.1 | 0.30 |
| Parma | 7_F. |  | 5 years | 22.41 | 0.26 |
| Velia | T375. |  | 5 years | 17.68 | 0.21 |
| Paks | 1164. |  | 5-6 years | 16.7 | 0.26 |
| Velia | T390. |  | 5-6 years | 15.18 | 0.22 |
| Bologna | 1_M. |  | 5.8 years | 16.94 | 0.22 |
| Beli Manastir | G20. |  | 5.5-6 years | 22.14 | 0.20 |
| Bologna | BO6_F. |  | 5 years and 10 months | 19.61 | 0.22 |
| Bologna | 11_F. |  | 6 years | 17.55 | 0.21 |
| PerkataNyuli | 435. | Childhood | 6-7 years | 16.31 | 0.27 |
| Velia | T333. |  | 6-7 years | 22.81 | 0.23 |
| Bologna | 6_M. |  | 7 years | 16.85 | 0.28 |
| PerkataNyuli | 1575. |  | 6-8 years | 24 | 0.26 |
| Beli Manastir | G31. |  | 7-8 years | 24.37 | 0.18 |
| Paks | 1865. |  | 7-8 years | 25.51 | 0.19 |
| PerkataNyuli | 2123. |  | 7-8 years | 26.66 | 0.22 |
| PerkataNyuli | 752. |  | 7-8 years | 18.18 | 0.20 |
| Velia | T320. |  | 7-8 years | 28.38 | 0.21 |
| Bologna | 40_M. |  | 9 years | 13.36 | 0.22 |
| Velia | T138. |  | 9-10 years | 34.78 | 0.18 |

Table S3 – Individuals’ averages for Tb.N, Tb.Sp and Tb.Th

| Name | Age Class | Age at death | Mean Tb.N | Mean Tb.Sp (SD) | Mean Tb.Th (SD) |
| --- | --- | --- | --- | --- | --- |
| VeliaT305. | Neonates  and toddlers | 0-3 months | 1.98 | 0.38(0.22) | 0.13 (0.03) |
| NF821369. |  | 8 weeks | 1.73 | 0.48(0.20) | 0.10 (0.03) |
| NF821045. |  | 0-6 months | 2.22 | 0.32(0.17) | 0.13 (0.05) |
| VeliaT300. |  | 0-6 months | 1.15 | 0.73(0.36) | 0.14 (0.03) |
| VeliaT441. |  | 0-6 months | 1.86 | 0.41(0.21) | 0.13 (0.03) |
| NF821051. |  | 7.5 months | 1.12 | 0.68(0.33) | 0.21 (0.07) |
| VeliaT442. |  | 6-9 months | 1.57 | 0.49(0.24) | 0.14 (0.03) |
| VeliaT368. |  | 0.75-1 year | 1.56 | 0.51(0.23) | 0.13 (0.03) |
| VeliaT289. |  | 9-12 months | 1.36 | 0.58(0.25) | 0.16 (0.04) |
| BO58_M. |  | 11 months | 1.53 | 0.47(0.18) | 0.18 (0.05) |
| BO60_F. |  | 11 months | 1.38 | 0.58(0.27) | 0.14 (0.04) |
| NF820614. |  | 12 months | 1.06 | 0.81(0.30) | 0.13 (0.02) |
| VeliaT415. | Early Infancy | 1-1.5 | 1.41 | 0.53(0.23) | 0.17 (0.05) |
| VeliaT434. |  | 1-1.5 | 1.28 | 0.58(0.20) | 0.20 (0.04) |
| BO14_M. |  | 1yrs and 5 mths | 1.22 | 0.68(0.24) | 0.14 (0.04) |
| NF821014. |  | 1.5 | 1.20 | 0.58(0.25) | 0.25 (0.07) |
| NF821046. |  | 1.5 | 1.14 | 0.69(0.33) | 0.19 (0.05) |
| PerkataNyuli655. |  | 1-3. | 0.90 | 0.92(0.50) | 0.19 (0.04) |
| VeliaT286. |  | 1.5-2 | 1.23 | 0.63(0.33) | 0.018 (0.05) |
| BO14_F. |  | 1.9 | 1.30 | 0.61(0.23) | 0.16 (0.05) |
| BO48_F. |  | 3 | 1.22 | 0.67(0.27) | 0.15 (0.05) |
| NF820683. |  | 3 | 1.11 | 0.69(0.28) | 0.21 (0.06) |
| NF821026. |  | 2 | 0.91 | 0.92(0.53) | 0.18 (0.05) |
| NF821207. |  | 2 | 0.95 | 0.87(0.35) | 0.19 (0.05) |
| NF821214. |  | 3 | 1.04 | 0.81(0.36) | 0.15 (0.04) |
| PerkataNyuli516. |  | 1.5-3 | 1.08 | 0.70(0.27) | 0.23 (0.08) |
| IlokG22. |  | 2-3. | 1.21 | 0.68(0.26) | 0.15 (0.04) |
| NF821069. |  | 2.5 | 1.19 | 0.64(0.30) | 0.20 (0.05) |
| NF821113. |  | 2.5 | 1.11 | 0.72(0.23) | 0.18 (0.05) |
| VeliaT411. |  | 2-3. | 1.18 | 0.64(0.24) | 0.21 (0.05) |
| BeliManastirG6. |  | 2-3.5 | 1.25 | 0.61(0.30) | 0.19 (0.06) |
| PerkataNyuli639. |  | 2.5-3 | 1.07 | 0.72(0.26) | 0.21 (0.06) |
| BO7_M. |  | 2.9 | 1.12 | 0.71(0.33) | 0.19 (0.06) |
| PerkataNyuli3421. |  | 2.5-3.5 | 1.13 | 0.67(0.26 | 0.21 (0.04) |
| NF819938. | Late Infancy | 3 | 1.18 | 0.68(0.25) | 0.17 (0.05) |
| IlokG70. |  | 3-4 years | 1.24 | 0.65(0.28) | 0.16 (0.05) |
| IlokG72. |  | 3-4. | 1.59 | 0.49(0.21) | 0.14 (0.04) |
| Paks1166. |  | 3-5. | 0.94 | 0.79(0.60) | 0.28 (0.08) |
| Paks997. |  | 3-5. | 1.25 | 0.61(0.22) | 0.19 (0.04) |
| VeliaT209. |  | 4 | 1.11 | 0.70(0.28) | 0.20 (0.05) |
| VeliaT342. |  | 4 | 1.15 | 0.67(0.26) | 0.20 (0.05) |
| Paks1846. |  | 4-5. | 1.07 | 0.68(0.25) | 0.25 (0.07) |
| PerkataNyuli4263. |  | 4-5. | 1.15 | 0.62(0.21) | 0.25 (0.09) |
| BO4_F. |  | 5 | 1.23 | 0.62(0.28) | 0.19 (0.06) |
| BO5_F. |  | 5 | 1.29 | 0.61(0.25) | 0.17 (0.05) |
| NF821012. |  | 5 | 1.01 | 0.81(0.34) | 0.17 (0.05) |
| PARMA7_F. |  | 5 | 1.22 | 0.60(0.26) | 0.22 (0.07) |
| VeliaT375. |  | 5 | 1.27 | 0.60(0.20) | 0.19 (0.04) |
| Paks1164. |  | 5-6. | 1.24 | 0.61(0.24) | 0.19 (0.04) |
| VeliaT390. |  | 5-6. | 0.96 | 0.81(0.31) | 0.23 (0.07) |
| BO1_M. |  | 5.8 | 1.02 | 0.79(0.39) | 0.19 (0.06) |
| BeliManastirG20. |  | 5.5-6 | 1.25 | 0.60(0.24) | 0.20 (0.08) |
| BO6_F. |  | 5.10mthd | 1.49 | 0.50(0.20) | 0.17 (0.05) |
| BO11_F. |  | 6 | 1.27 | 0.60(0.25) | 0.19 (0.06) |
| PerkataNyuli435. | Childhood | 6-7. | 1.07 | 0.74(0.33) | 0.20 (0.03) |
| VeliaT333. |  | 6-7. | 1.08 | 0.68(0.28) | 0.24 (0.06) |
| BO6_M. |  | 7 | 1.29 | 0.60(0.23) | 0.18 (0.05) |
| PerkataNyuli1575. |  | 6-8. | 0.98 | 0.76(0.41) | 0.26 (0.09) |
| BeliManastirG31. |  | 7-8. | 1.35 | 0.57(0.26) | 0.17 (0.06) |
| Paks1865. |  | 7-8. | 1.01 | 0.71(0.33) | 0.28 (0.09) |
| PerkataNyuli2123. |  | 7-8. | 1.14 | 0.61(0.27) | 0.26 (0.08) |
| PerkataNyuli752. |  | 7-8. | 1.16 | 0.66(0.25) | 0.21 (0.04) |
| VeliaT320. |  | 7-8. | 1.22 | 0.56(0.23) | 0.26 (0.07) |
| BO40_M. |  | 9 | 1.23 | 0.65(0.28) | 0.16 (0.04) |
| VeliaT138. |  | 9-10. | 1.14 | 0.56(0.23) | 0.31 (0.09) |


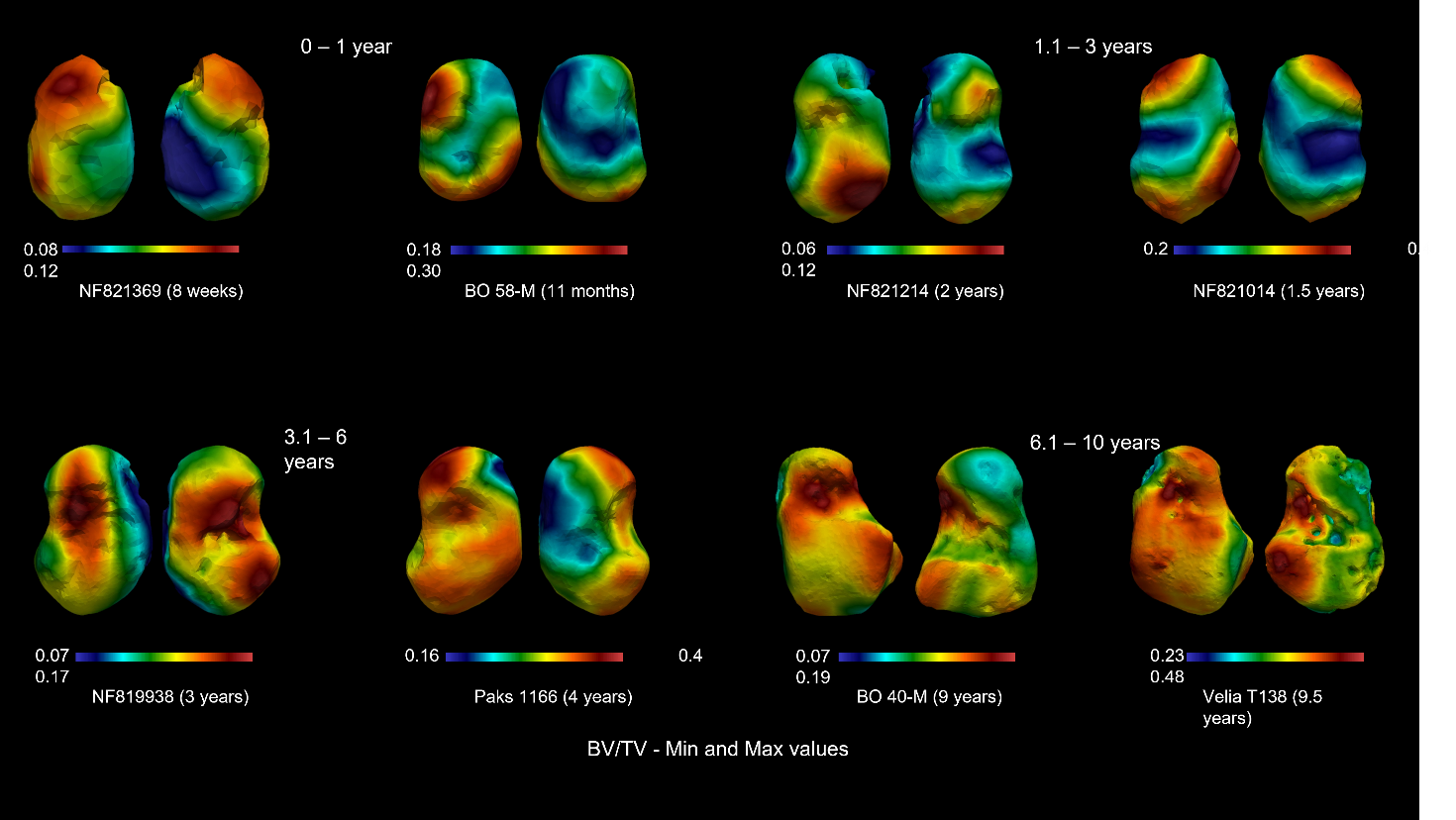


Figure S4 - BV/TV ranges within each age group.


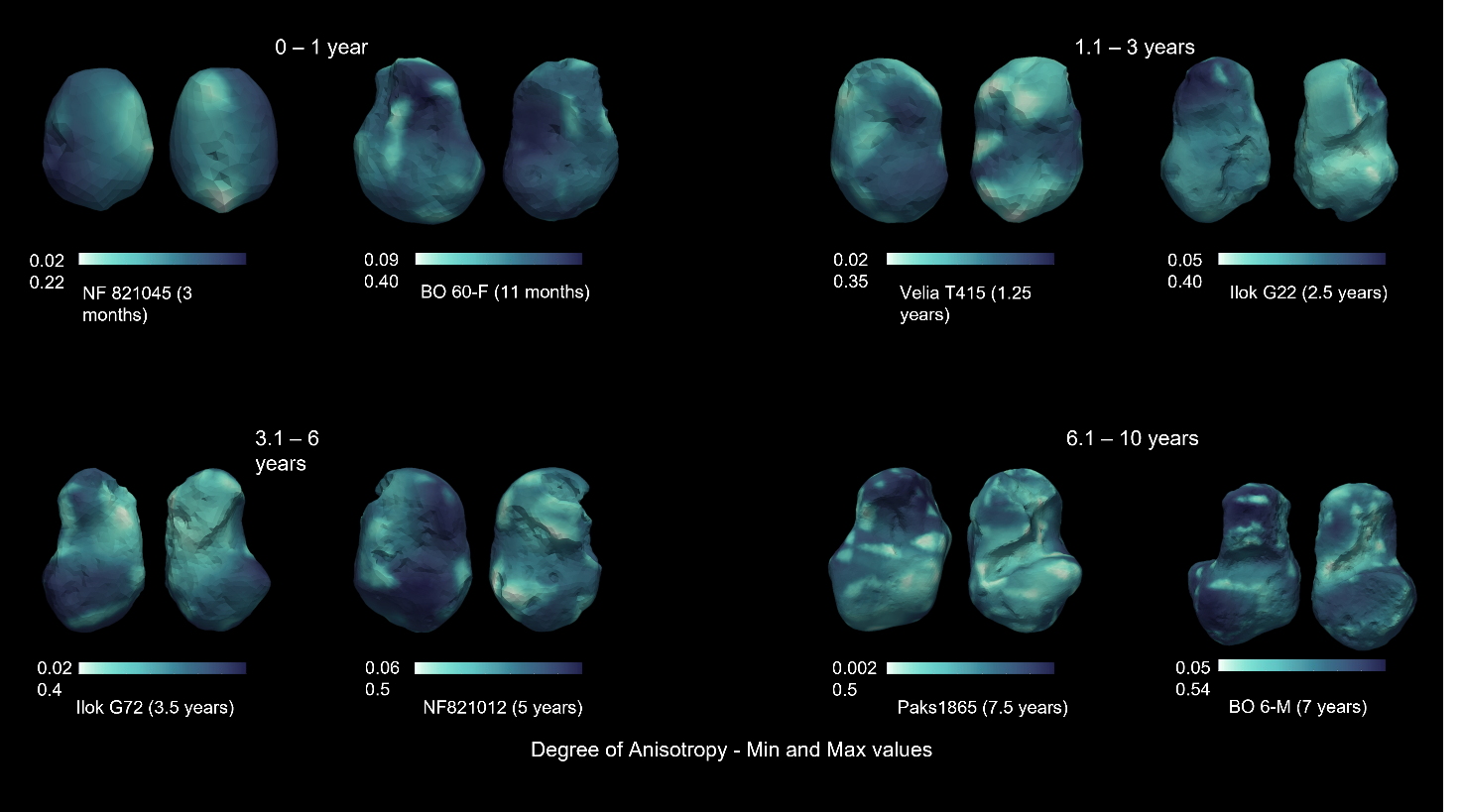


Figure S5 - DA ranges within each age group.


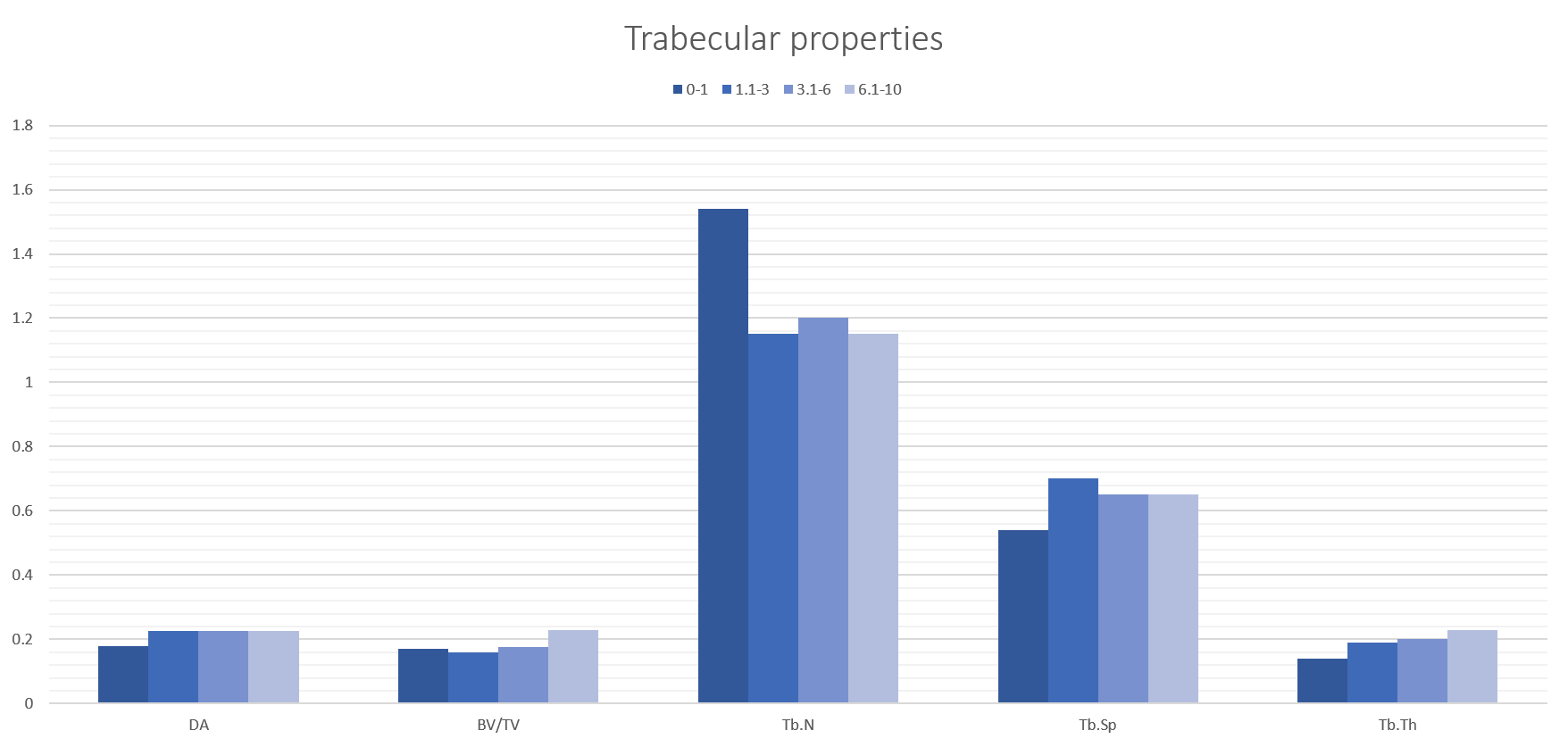


Figure S6 – Mean values of trabecular properties are represented for each age group.
